# NOX2-mediated reactive oxygen species are double-edged swords in focal cerebral ischemia in mice

**DOI:** 10.1186/s12974-022-02551-6

**Published:** 2022-07-14

**Authors:** Ye Yingze, Jian Zhihong, Jin Tong, Li Yina, Zeng Zhi, Zhang Xu, Xiong Xiaoxing, Gu Lijuan

**Affiliations:** 1 Central Laboratory, Renmin Hospital of Wuhan University, Wuhan, 430060 China; 2 Department of Neurosurgery, Renmin Hospital of Wuhan University, Wuhan, 430060 China; 3 Department of Pathology, Renmin Hospital of Wuhan University, Wuhan, 430060 China

**Keywords:** Ischemic stroke, NADPH, ROS, Autophagy, NLRP3 inflammasome, Angiogenesis

## Abstract

**Background:**

Reactive oxygen species (ROS) often promote acute brain injury after stroke, but their roles in the recovery phase have not been well studied. We tested the hypothesis that ROS activity mediated by NADPH oxidase 2 (NOX2) contributes to acute brain injury but promotes functional recovery during the delayed phase, which is linked with neuroinflammation, autophagy, angiogenesis, and the PI3K/Akt signaling pathway.

**Methods:**

We used the NOX2 inhibitor apocynin to study the role of NOX2 in brain injury and functional recovery in a middle cerebral artery occlusion (MCAO) stroke mouse model. Infarct size, neurological deficits and behavior were evaluated on days 3, 7, 10 and 14 after reperfusion. In addition, dynamic NOX2-induced ROS levels were measured by dihydroethidium (DHE) staining. Autophagy, inflammasomes, and angiogenesis were measured by immunofluorescence staining and western blotting. RNA sequencing was performed, and bioinformatics technology was used to analyze differentially expressed genes (DEGs), as well as the enrichment of biological functions and signaling pathways in ischemia penumbra at 7 days after reperfusion. Then, Akt pathway-related proteins were further evaluated by western blotting.

**Results:**

Our results showed that apocynin injection attenuated infarct size and mortality 3 days after stroke but promoted mortality and blocked functional recovery from 5 to 14 days after stroke. DHE staining showed that ROS levels were increased at 3 days after reperfusion and then gradually declined in WT mice, and these levels were significantly reduced by the NOX2 inhibitor apocynin. RNA-Seq analysis indicated that apocynin activated the immune response under hypoxic conditions. The immunofluorescence and western blot results demonstrated that apocynin inhibited the NLRP3 inflammasome and promoted angiogenesis at 3 days but promoted the NLRP3 inflammasome and inhibited angiogenesis at 7 and 14 days after stroke, which was mediated by regulating autophagy activation. Furthermore, RNA-Seq and Kyoto Encyclopedia of Genes and Genomes (KEGG) analysis indicated that apocynin injection resulted in PI3K–Akt signaling pathway enrichment after 7 days of MCAO. We then used an animal model to show that apocynin decreased the protein levels of phosphorylated PI3K and Akt and NF-κB p65, confirming that the PI3K–Akt–NF-κB pathway is involved in apocynin-mediated activation of inflammation and inhibition of angiogenesis.

**Conclusions:**

NOX2-induced ROS production is a double-edged sword that exacerbates brain injury in the acute phase but promotes functional recovery. This effect appears to be achieved by inhibiting NLRP3 inflammasome activation and promoting angiogenesis via autophagy activation.

**Supplementary Information:**

The online version contains supplementary material available at 10.1186/s12974-022-02551-6.

## Introduction

Excessive reactive oxygen species (ROS) production after ischemia/reperfusion contributes to acute brain injury after stroke. Nicotinamide adenine dinucleotide phosphate oxidase (NADPH oxidase, NOX) is the main enzyme responsible for ROS generation [1]. NOX2, formerly gp91phox, is the most widely distributed NOX isoform in the central nervous system (CNS) and has been extensively studied in various CNS diseases, including cerebral ischemia. Previous studies have demonstrated that NOX2 is upregulated in the ischemic penumbra and core after ischemic stroke onset [2–4], and NOX2-mediated ROS production promotes brain injury poststroke [3]. On the other hand, the NOX2 inhibitor apocynin reduces ROS generation and ameliorates neuronal death and cerebral injury induced by stroke both in vitro and in vivo [5]. Therefore, NOX2 promotes acute brain injury after stroke. Considering these detrimental effects of NOX2, other studies have suggested that NOX2 may also play beneficial roles in certain pathological conditions. Genetic deficiency or system inhibition of NOX2 increased hypoxia/ischemia-induced infarct and brain injury in newborn mice 7 days after stroke [6]. In addition, NOX2 is beneficial in the peripheral vasculature after hind limb ischemic injury by increasing capillary density and perfusion and contributing to the proliferation of endothelial cells [7]. However, whether NOX2 has dual roles in brain injury after stroke remains unknown.

The excessive ROS generated by NOX2 can cause inflammasome activation, which mediates the inflammatory response. NLRP3 inflammasomes, which are members of the NOD-like receptor family, are multiprotein complexes that serve as platforms for caspase activation and regulate cytokine maturation, inflammation and cell death (pyroptosis) and are also the most extensively studied inflammasomes in CNS diseases [8, 9]. Furthermore, NLRP3 inflammasome activation has been reported in cerebral ischemia/reperfusion injury (CIRI) models, as well as in stroke patients [10–12]. Overall, NOX2 appears to play an important role in the activation of ROS-dependent NLRP3 inflammasomes [11].

Moreover, NOX2 promotes angiogenesis. NOX2 was reported to be localized in angiogenic blood vessels in the rat brain up to 7 days poststroke with reperfusion [13]. In addition, NOX2-derived ROS are thought to act as effectors of angiogenesis and may function in brain recovery and regeneration after cerebral ischemia [14]. Furthermore, ROS exert biphasic effects characterized by the promotion of oxidative injury in the early phase and increasing angiogenesis in the late phase in cerebral ischemia.

Autophagy, which is an evolutionarily conserved process, eliminates damaged proteins and organelles that accumulate in eukaryotes [15]. NOX serves as a gate keeper to regulate the activation of autophagy based on cell type and cellular conditions [16, 17], and NOX-mediated effects on autophagy are ROS-dependent [18]. The autophagy machinery is linked with the inflammasome, and autophagy inhibits NLRP3 inflammasome activation, while autophagy inhibition or deficiency promotes NLRP3 inflammasome activity and the release of IL-1β and IL-18 [19, 20]. In addition, autophagy induction can promote vascular growth, while autophagy inhibition blocks angiogenesis, and this process is mediated by ROS production [21]. Furthermore, both autophagy and angiogenesis are associated with the PI3K/Akt/NF-kB pathway. However, whether NOX2 regulates autophagy, the NLRP3 inflammasome and angiogenesis in ischemic stroke is still poorly understood, and whether the PI3K/Akt/NF-kB pathway is involved in these processes is unknown.

In this study, we first tested the hypothesis that NOX2 plays dual roles in acute brain injury and delayed functional recovery after ischemic stroke. We found that NOX2-mediated ROS production as necessary and beneficial for brain functional recovery. In addition, we investigated the mechanisms of the distinctive effect of NOX2–ROS on the ischemic brain during different periods of stroke and showed that these effects were linked with dynamic autophagy activation, which regulated the NLRP3 inflammasome and angiogenesis through the PI3K/Akt pathway.

## Materials and methods

### Animals

Male wild-type C57BL/6J mice (*n* = 300; 25–30 g, 8–10 weeks) were purchased from Hubei Experimental Animal Research Center (Hubei, China; Nos. 43004700018817 and 43004700020932). All animal experimental protocols were approved by the Animal Experimentation Ethics Committee of Wuhan University (No. WDRM-20170504) and were conducted according to the Animal Care and Use Committee guidelines of Renmin Hospital of Wuhan University. Animals were housed in a room with controlled humidity (65 ± 5%) and temperature (25 ± 1 °C) under a 12/12-h light/dark cycle with free access to food and water for at least 1 week before the experiments.

### Drug administration

Apocynin (178385, Sigma, St Louis, MO, USA) was dissolved in dimethyl sulfoxide and then diluted with sterile saline to the desired concentrations. Different concentrations of apocynin were intraperitoneally (i.p.) administered to the animals at a dose of 2.5 mg/kg 8 h after reperfusion as described by Qin et al. [22]. 3-MA (M9281, Sigma-Aldrich, St Louis, MO, USA) was dissolved in physiological saline and administered (15 mg/kg, i.p.) 1 and 3 h after occlusion [23]. Rapamycin (553210, Sigma-Aldrich, St Louis, MO, USA) was stored at room temperature and dissolved in DMSO with gentle heating to yield a clear, colorless solution and then injected (10 mg/kg, i.p.) immediately after MCAO [24].

### MCAO model

The MCAO model was established as previously described [25]. In brief, C57BL/6J wild-type mice were anesthetized with 5% isoflurane in O_2_ by a facemask, followed by ligation of the left middle cerebral artery with a 6-0 monofilament (Doccol Corp., Redlands, CA, USA). After 1 h of occlusion, the monofilament was removed to initiate reperfusion. A homeothermic heating pad was used to monitor and stabilize the body temperature at 37 ± 0.5 °C. The same procedure, but without monofilament ligation, was performed on sham-operated mice.

### Infarct volume measurement

The mice were deeply anesthetized, euthanized with an overdose of isoflurane and decapitated 3, 7 and 14 days after MCAO. The brains were collected after transcranial perfusion with saline followed by 4% paraformaldehyde. After postfixation with 4% paraformaldehyde for 72 h, brain tissues were cut into 50-µm coronal sections, dipped in 0.1% cresyl violet solution for 30 min, and then rinsed in distilled water. The stained sections were fixed by serial dehydration in ethanol (70%, 90%, 100%) and xylene. The infarct volume was measured and analyzed by a blinded observer using ImageJ v1.37 (NIH, Bethesda, MA, USA), as described previously [26, 27], and the data were normalized and are presented as a percentage of the nonischemic hemisphere to correct for edema.

### Assessment of neurological deficits

Neurological deficit scores were evaluated 3, 7 and 14 days after MCAO as described previously [28]. The score ranged from 0 (without observable neurological deficit) to 4 (no spontaneous motor activity and loss of consciousness).

### Rotarod test

Accelerating rotarod (SD Instruments, San Diego, CA) instruments were used to test the motor coordination of the mice. Each mouse was placed on a 2.75 cm diameter rotating rod every other day before MCAO onset for a total of 9 training days, and the rotation speed of the rod increased from 5 to 10 rpm every 5 min. The time between the beginning of the mouse staying on the rod and falling from the rod was determined up to a maximum duration of 300 s. After the training period, MCAO surgery was conducted, and rotarod testing was performed by a blinded observer on Days 0, 3, 7, 10, and 14 poststroke. The scores were calculated by averaging the three repetitive time records of each mouse each day.

### Ischemic core and penumbra segmentation

The separation of ischemic core and penumbra was performed as previously descriptions with minor revision based on the average infarct volume measured by TTC or cresyl violet staining in our pilot experiments and the heterogeneity of penumbra as a dynamic and changeable process over time [29–33]. Mice were deeply anesthetized and euthanized with an overdose of isoflurane, the brain tissue was quickly collected and put on ice and the olfactory bulb, cerebellum and low brain stem were removed. Firstly, the brain tissue was cut on a coronal plane 3 mm backward from the top of the frontal lobe into three slices with thickness of 3 mm (section 1), 4 mm (section 2) and 3 mm (section 3), respectively. Then, the section 2 was taken out and the midline was identified between the ipsilateral (left) and contralateral (right) hemispheres. Next, a sagittal cut 1.5 mm far from the midline was conducted from top to bottom on the ipsilateral hemisphere, and a transverse diagonal cut was also made at around “1 o'clock” position. Thus, the tissue outside “1 o'clock” was the infarct core area, and the cortical tissue between sagittal cut and “1 o'clock” was the ischemic penumbra (Additional file 1: Fig. S1).

### High-throughput RNA sequencing (RNA-Seq)

Total RNA was extracted from the penumbral region of the ischemic hemisphere on Day 7 after MCAO using TRIzol reagent (Invitrogen, Carlsbad, CA, USA). Subsequently, total RNA was quantified by using a NanoDrop ND-1000, and 1 ~ 2 μg of total RNA was used to construct the RNA-Seq libraries. mRNA was enriched using the NEBNext® Poly(A) mRNA Magnetic Isolation Module (New England Biolabs). Libraries were then constructed using a KAPA Stranded RNA-Seq Library Prep kit (Illumina) according to the manufacturer's protocol. The sequencing library was examined by an Agilent 2100 Bioanalyzer using an Agilent DNA 1000 chip kit (Agilent, part # 5067-1504). All samples were sequenced on an Illumina HiSeq 4000 with 150 bp paired-end reads. After quality control was performed, the raw sequencing data were aligned to the mouse genome (GRCm38) using Hisat2 software. Finally, differentially expressed genes were defined as those with a fold change ≥ 1.5 and *p* value ≤ 0.05. Cluster analysis was performed using Cluster 3.0 software. Gene Ontology (GO) biological process analysis was performed using DAVID, and KEGG was used for pathway analysis.

### Measurement of ROS

To assess ROS production, the brain was carefully and quickly isolated, cut into 4.0 μm sections and placed on chilled microscope slides. The samples were incubated in physiological saline containing 10 μmol dihydroethidium (DHE; Sigma–Aldrich) for 30 min at 37 °C in the dark. The brain sections were washed twice with PBS and placed under an automatic fluorescence microscope (BX63, Olympus Optical Ltd, Tokyo, Japan).

### Immunofluorescence staining

Immunofluorescence analysis was performed as previously described [26]. Ischemic and sham-operated mice were euthanized and perfused with cold PBS, followed by fixation with 4% paraformaldehyde for 2 days. The ischemic brains were cut into 50-μm sections, and the free-floating slices were blocked with 0.1 M PBS containing 5% fetal bovine serum and 0.3% Triton X for 1 h at room temperature. After being washed, the slices were incubated at 4 °C overnight with the following primary antibodies: anti-NLRP3 (1:200; ab4207, Cell Signaling Technology, Boston, USA), anti-LC3B (1:200; ab104224, Abcam, Cambridge, England), and anti-CD31 (1:100, GB11224, Servicebio, Wuhan, China). The slices were then rinsed and incubated with an Alexa 594-conjugated antibody (1:200; ANT030, Millipore, Billerica, MA) or an Alexa 488-conjugated antibody (1:200; ANT024, Millipore, Billerica, MA) for 2 h at room temperature. After being thoroughly rinsed, the nuclei were stained with DAPI (94010, Vector Laboratories, Burlingame, CA, USA). All slices were photographed using a confocal fluorescence microscope (BX63, Olympus Optical Ltd, Tokyo, Japan). The number of immunoreactive cells in predefined areas was quantified using ImageJ software (Media Cybernetics Inc., Rockville, MD, USA). Six different fields for each mouse and six mice for each group were counted. All counts were conducted by blinded observers.

### Western blot analysis

Western blotting was carried out as previously described [25]. Cortical sections 1.0 to 2.0 mm from ipsilateral brain tissue were harvested and homogenized in cold RIPA buffer (C1053, Applygen, Beijing, China) containing a protease inhibitor cocktail (G2006, Servicebio, Wuhan, China). The homogenates were centrifuged at 4 °C at 10,000×*g* for 30 min, and then the supernatants were harvested. Protein levels were determined with a BCA kit (G2026, Servicebio, Wuhan, China). Protein samples (20 μl/lane) were separated by electrophoresis on 4–15% sodium dodecyl sulfate–polyacrylamide gels and then transferred onto PVDF membranes (Millipore, Billerica, MA, USA). The membranes were then placed into 5% nonfat milk in PBS/0.1% Tween and blocked for 1 h, followed by incubation overnight with mouse anti-p62 (1:300, #16177, Cell Signaling Technology, Boston, USA), anti-NLPR3 (1:1000; ab4207, Cell Signaling Technology, Boston, USA), anti-ASC (1:1000; 67824, Cell Signaling Technology, Boston, USA), anti-CL-caspase-1 (1:500; 89332, Cell Signaling Technology, Boston, USA), anti-HMGB1 (1:1000; ab18256, Abcam, Cambridge, England), anti-IL-1β (1:500; ab8320, Abcam, Cambridge, England), anti-NOX2 (1:1000; ab18256, Abcam, Cambridge, England), anti-Beclin-1 (1:1000; ab18256, Abcam, Cambridge, England), anti-PI3K (1:1000; ab8378, Abcam, Cambridge, England), anti-p-PI3K (1:1000; ab8378, Abcam, Cambridge, England), anti-Akt (1:1000; ab8378, Abcam, Cambridge, England), anti-p-Akt (1:1000; ab8378, Abcam, Cambridge, England), anti- NF-κB p65 (1:1000; ab8378, Abcam, Cambridge, England), anti-phosphorylated NF-κB p65 (1:1000; 3033S, Cell Signaling Technology, Boston, USA) and anti-VEGF (1:1000; 2042744, Millipore, Billerica, MA) at 4 °C. After being washed with PBS/0.1% Tween, the membrane was incubated with IRDye-labeled secondary antibodies (1:10,000; c60405-05, Li-Cor Bioscience, USA) at room temperature for 1–2 h. Images were acquired with an Odyssey western blot analysis system (LI-COR, Lincoln, NE, USA). The relative band intensity was calculated using Quantity One v4.6.2 software (Bio–Rad Laboratories, Hercules, USA) and then normalized to the GAPDH loading control. These experiments were performed three times.

### Statistical analysis

The results are shown as the mean ± SD and were analyzed using SPSS 22.0 software. The specific statistical methods are described in each figure legend. One-way ANOVA was performed when comparing different groups, whereas differences in two groups were evaluated using unpaired Student's t tests. *P* ≤ 0.05 denotes significant changes.

## Results

### Pharmacological inhibition of NOX2 protects against infarction but inhibits functional recovery after stroke

**Fig. 1:**
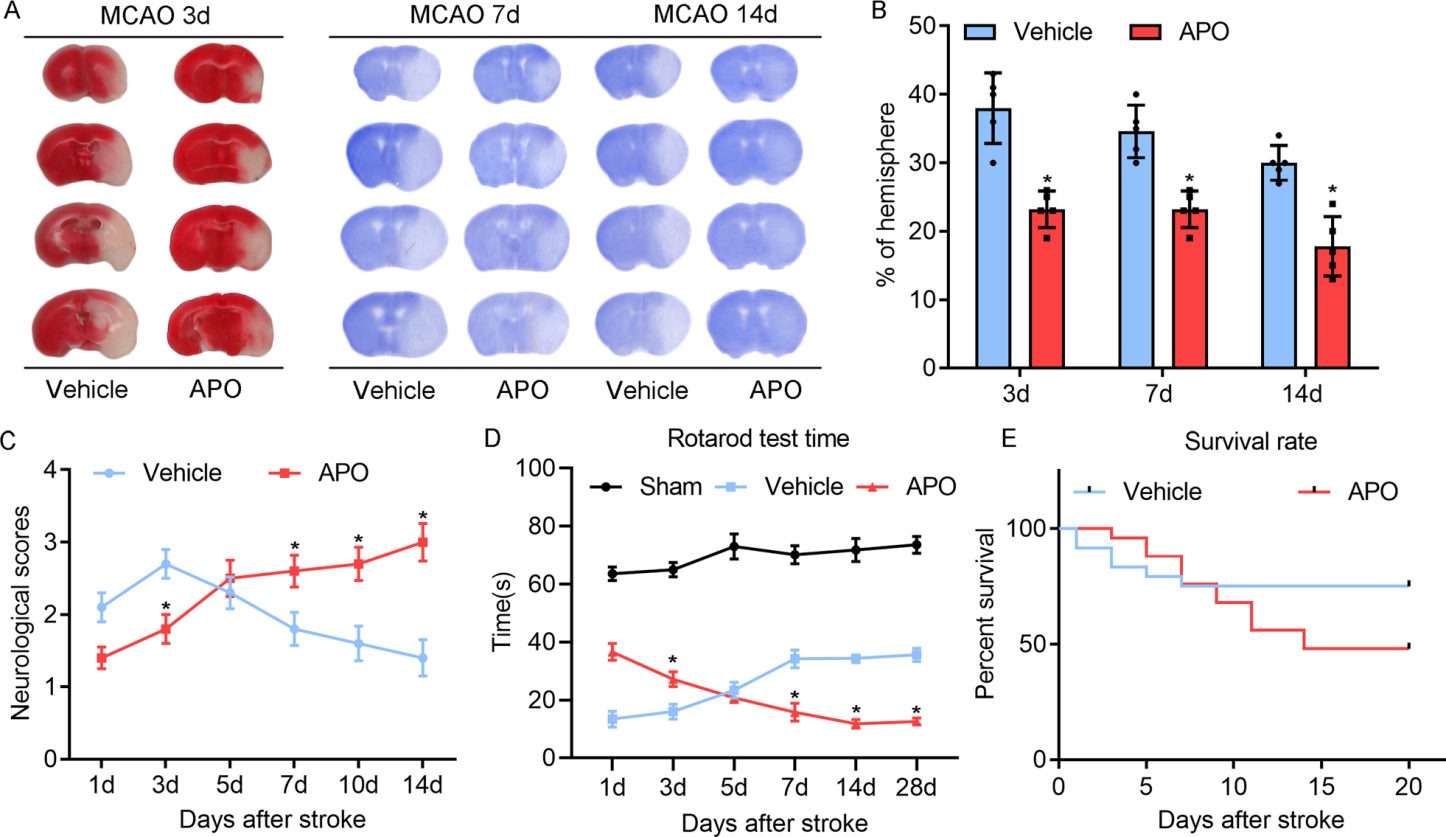
Effect of apocynin on brain injury and neurological deficit scores after MCA occlusion at different time points. The multilateral infarct volume was measured 3, 7 and 14 days after stroke onset by using Cresyl Violet staining and TTC staining, and the results were normalized to the heterolateral hemisphere to correct for edema and are expressed as a percentage. Infarcted areas are lighter (white) in color. **A** Apocynin reduced the infarct size in MCAO model mice 3, 7 and 14 days poststroke, as shown by TTC staining and Cresyl Violet staining. **B** Ipsilateral infarct size was normalized to the contralateral hemicerebrum and is presented as a percentage. **C** Neurological scores were lower 3 days after stroke but higher 7 and 14 days after stroke in apocynin-treated mice than in the Vehicle group. **D** Rotarod test. **E** Survival rate. All graphs show the mean ± SD, *P < 0.05, **P < 0.01 vs. vehicle. *n* = 9–13/group

We first examined the effects of the NOX2 inhibitor apocynin on brain infarction and functional recovery. Three different doses of apocynin (1.5, 2.0 and 2.5 mg/kg) were studied. The results showed that 2.0 mg/kg apocynin reduced the infarct volume to approximately half that of the vehicle group; thus, this dose was selected for subsequent experiments (Additional file 2: Fig. S2). Then, we examined and compared the infarct volume by TTC staining at 3 days and Cresyl Violet staining at 7 and 14 days in WT mice and APO-treated mice subjected to MCAO. The results showed that the infarct volumes were significantly reduced in APO-treated mice compared with those in WT mice 3, 7 and 14 days after reperfusion (Fig. 1A, B). Although the neurological scores of APO-treated mice were lower within 3 days of stroke than those of WT mice, they worsened on Days 7 and 14 after stroke, when the neurological scores of WT animals had recovered to near normal levels (Fig. 1C). We then conducted a rotarod test to evaluate the effect of APO treatment on motor coordination. Similar to the neurological scores, both behavioral tests indicated that APO treatment improved behavioral performance on Day 3 but hindered functional recovery on Days 7 and 14 (Fig. 1D, E). Therefore, WT mice exhibited a significantly faster recovery than APO-treated mice.

### Stroke results in ROS production, which is NOX2-dependent

**Fig. 2:**
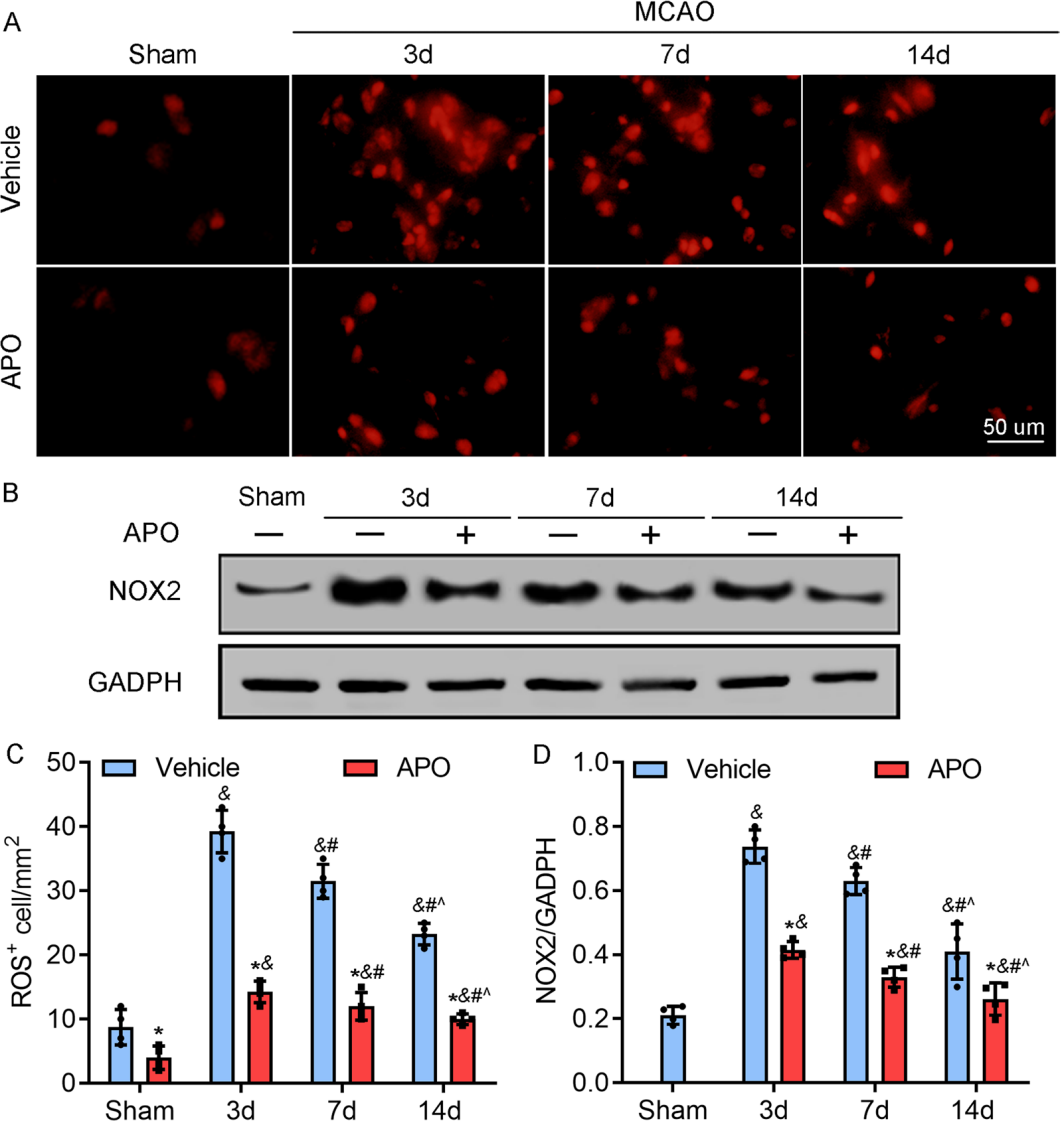
Dynamic changes in NOX2 and ROS during stroke. **A**, **C** The dynamic production of ROS was revealed by DHE staining and was closely dependent on the expression of NOX2, and the change in ROS was similar to the change in NOX2. Apocynin significantly inhibited ischemia/reperfusion-induced increases in ROS levels. **B**,** D** Representative western blots and quantitative analysis of the levels of NOX2 and the effects of apocynin on NOX2 expression in ischemic cortical tissue at the indicated time points during reperfusion. The expression of NOX2 was increased 3 days poststroke but gradually decreased to a level similar to that in the sham group over 14 days. Apocynin (2.0 mg/kg intraperitoneal injection, i.p.) markedly decreased the expression of NOX2 in ischemic cortical tissue. *P < 0.05 vs. the vehicle group, ^&^*P* < 0.05 vs. the sham group, ^#^*P* < 0.05 vs. the 3d MCAO group, ^^^*P* < 0.05 vs. the 7d MCAO group. Scale bar = 50 µm. All data are expressed as the mean ± SD and were analyzed by one-way ANOVA with Bonferroni's post hoc test. There were 5 animals in each group

As NOX2 is one of the main sources of ROS in the CNS, we evaluated NOX2 expression and ROS levels by using western blotting and DHE staining, respectively, to test whether the levels of ROS were related to NOX2. The results showed that the production of NOX2 and ROS were markedly increased at 3 days after stroke and gradually decreased between Day 3 and Day 14 (Fig. 2A and B). Furthermore, apocynin injection significantly suppressed the production of ROS and inhibited the expression of NOX2 (Fig. 2).

### Apocynin has different effects on angiogenesis and neuroinflammation on Day 3 than on Days 7 and 14 after stroke

**Fig. 3:**
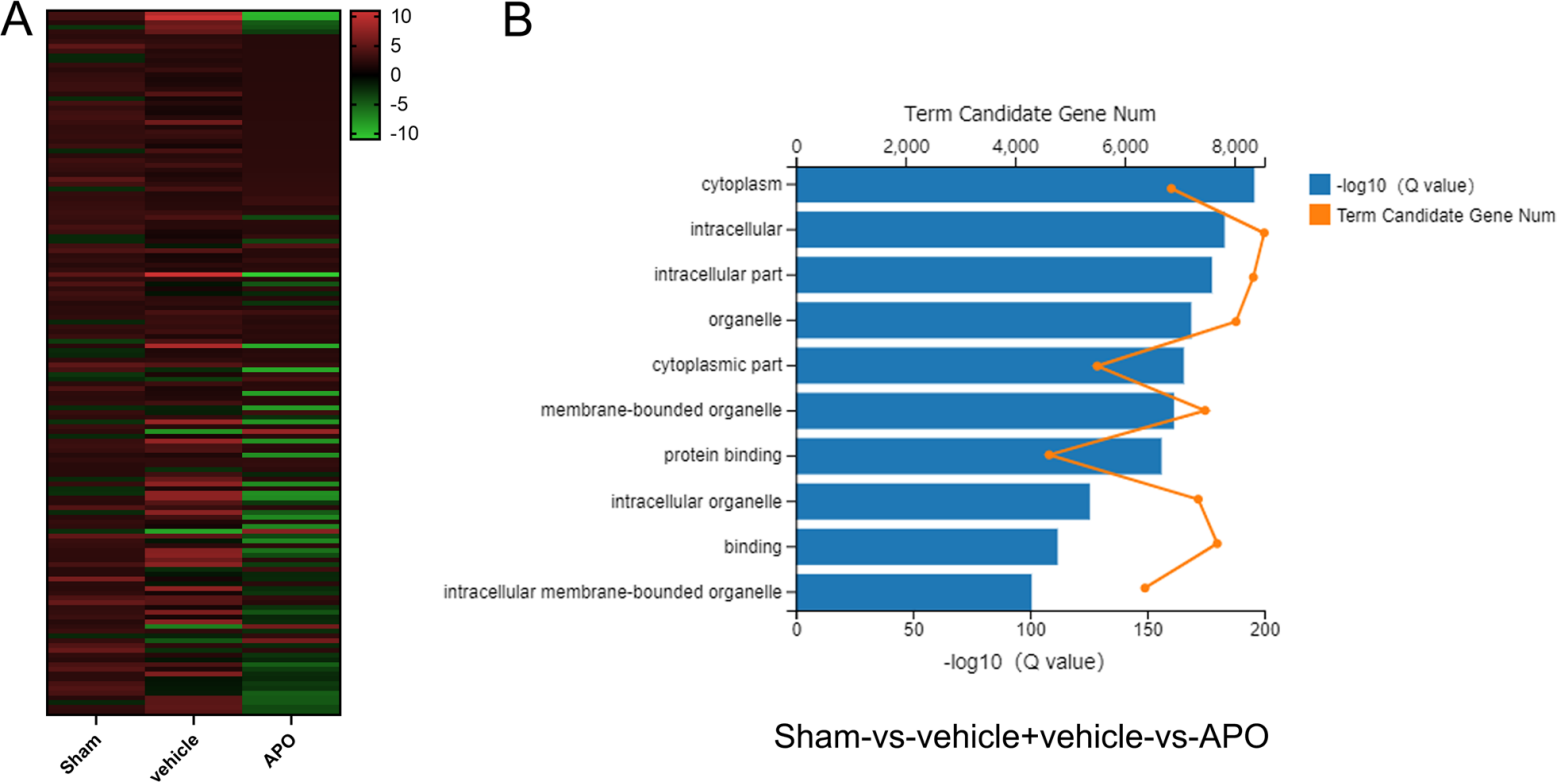
Transcriptome analysis identifies gene expression and functional classification during the recovery stage of ischemic stroke after treatment with apocynin. **A** Cluster analysis of the differentially expressed mRNAs in the sham, vehicle + MCAO and APO + MCAO groups based on RNA-Seq data. **B** GO enrichment analysis of the differentially expressed genes in the APO vs. the vehicle group compared to the sham vs. the vehicle group. The top 10 enriched biological processes are shown

**Fig. 4:**
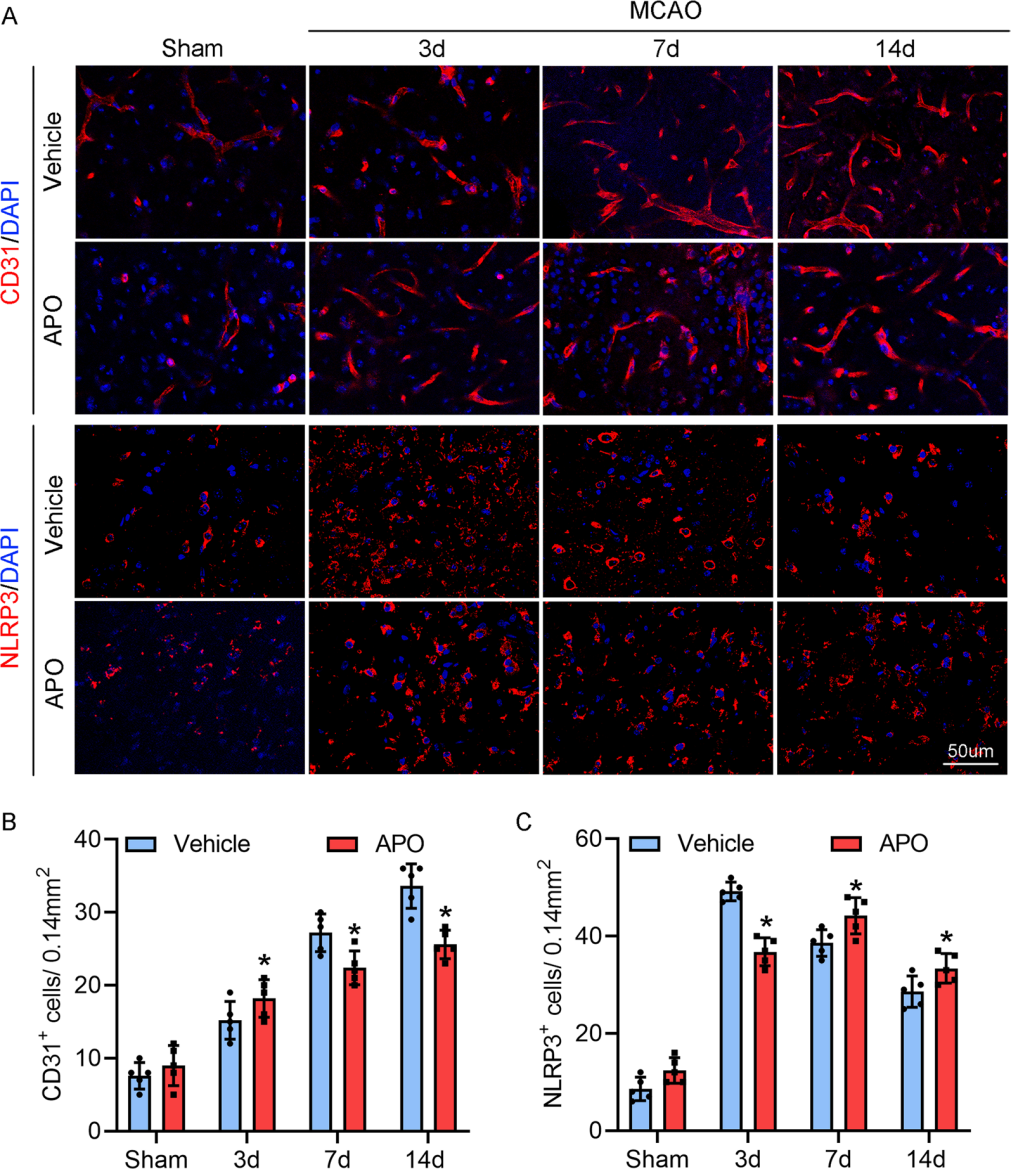
Effects of apocynin on the NLRP3 inflammasome and angiogenesis. **A**, **C** Apocynin significantly increased CD31 expression on Day 3 but gradually decreased CD31 staining from 7 to 14 days following stroke compared with the vehicle. **B**, **D** Apocynin significantly reduced NLRP3 expression on Day 3 but upregulated NLRP3 levels on Days 7 and 14 compared with those in the vehicle group. A, B Representative immunofluorescence images of CD31 and NLRP3 staining and DAPI counterstaining at 3, 7 and 14 days after stroke in the ischemic border. **C**, **D** Quantification of CD31- and NLRP3-positive cells in the ischemic border. *n* = 5/group. Scale bar = 50 μm. *P < 0.05 vs. the vehicle group. All data are expressed as the mean ± SD and were analyzed by one-way ANOVA with Bonferroni's post hoc test

In the core of the ischemic tissue, which is clinically defined as the area with regional cerebral blood flow < 20%, and outside the ischemic core, brain tissue is still partially perfused, although at a reduced rate. This region, which is referred to as the ischemic penumbra, is often defined by a reduced perfusion rate that is, however, greater than that observed in the ischemic core. In the treatment of ischemic stroke, it is urgent to rescue neurons in the penumbra region. To investigate the biphasic mechanism of ROS, we performed RNA sequencing on the penumbral zone of the ischemic cortex of MCAO mice on Day 7. Bioinformatics analysis showed that 1616 genes were upregulated and 106 genes were downregulated in ischemic tissue compared to those in the sham group. Furthermore, apocynin increased the expression of 284 genes and reduced the expression of 2019 genes compared to MCAO (Fig. 3A). GO analysis of the top 20 genes under ischemic conditions showed that these genes were closely related to several biological processes, such as the regulation of leukocyte migration, T cell activation, and the regulation of the innate immune response (Additional file 3: Fig. S3). Furthermore, the genes upregulated by NOX2 inhibition were associated with the following biological processes: neurotransmitter transport, neutrophil-mediated immunity and regulation of neuronal projection development (Additional file 4: Fig. S4). In addition, we obtained two datasets related to differentially expressed genes between the vehicle group and sham group, as well as the apocynin treatment group and vehicle group, and then compared these datasets to further analyze a new dataset of differentially expressed genes. Next, the enrichment of biological functions was assessed in this new dataset by GO analysis, and these genes were involved in membrane-bound organelles, intracellular organelles and cytoplasmic parts (Fig. 3B). Moreover, using this method, we also found that apocynin inhibited angiogenesis (data not shown). Angiogenesis and inflammatory reactions in the penumbra of the ischemic cortex are essential steps for improving recovery or exacerbating brain injury following ischemic stroke, respectively. We performed confocal analysis of CD31 (an angiogenesis biomarker) in samples and revealed that angiogenesis in the penumbra gradually increased from Day 3 to Day 14 following ischemic stroke (Fig. 4A, C). After apocynin treatment, CD31^+^ cells were further significantly increased on Day 3 after stroke compared with those in the vehicle group. However, apocynin markedly decreased CD31^+^ cells on day 7 and 14 following stroke compared with the vehicle (Fig. 4A, C).

The NLRP3 inflammasome reflects inflammatory status. The immunofluorescent staining results showed that NLRP3^+^ positive cells were increased at 3 days but gradually decreased from 7 to 14 days following stroke in animals without apocynin (Fig. 4B, D). However, apocynin treatment significantly reduced NLRP3 expression on Day 3 after stroke but gradually increased NLRP3 levels from 7 to 14 days (Fig. 4B, D).

### Autophagy is NOX2/ROS-dependent after stroke

**Fig. 5:**
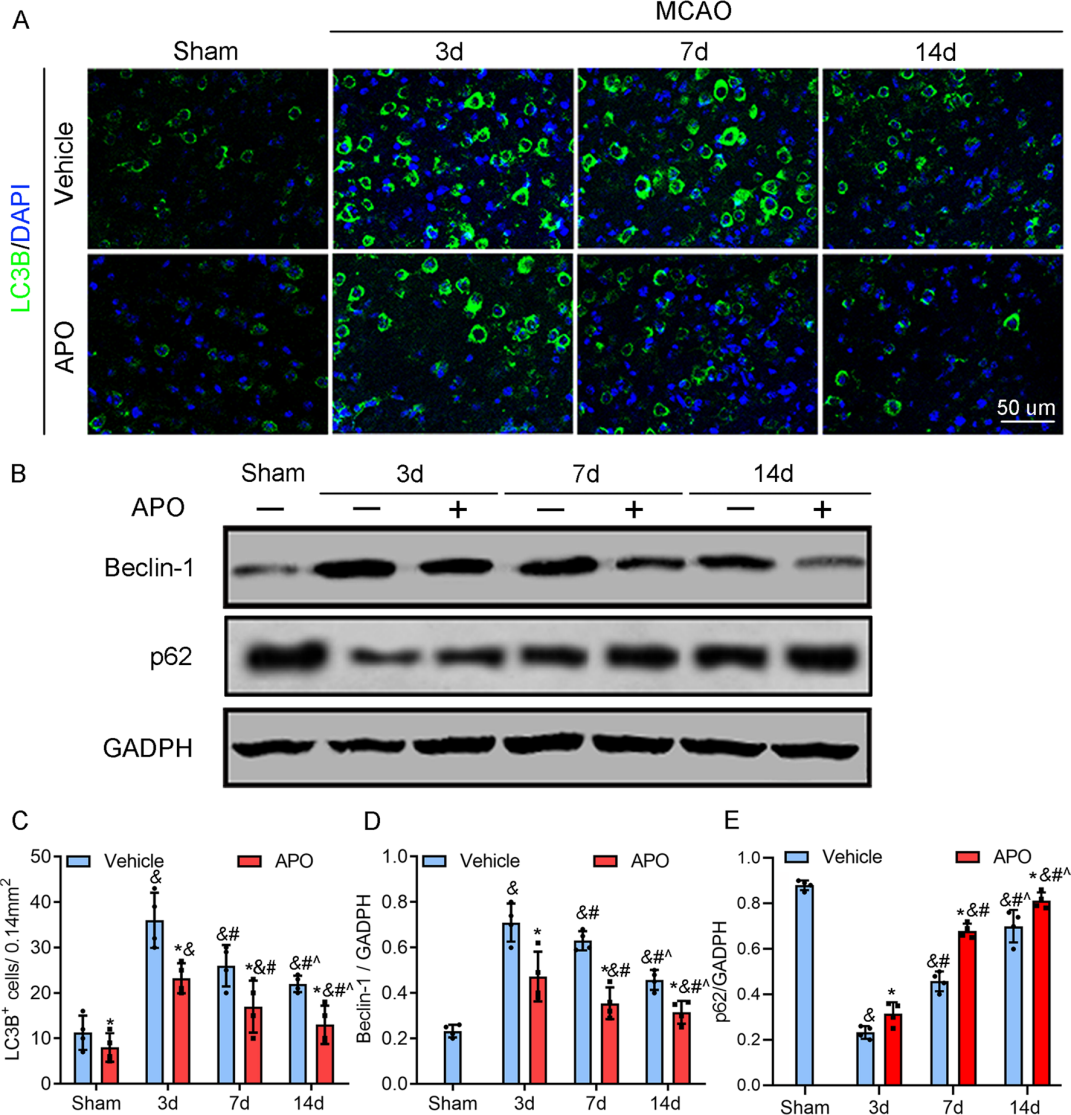
Dynamic changes in autophagy-related proteins during stroke. **A**, **C** Expression of LC3B was revealed by immunofluorescent staining and was increased on Day 3 and gradually decreased with increasing time, and the expression of LC3B was inhibited by apocynin in all groups, which had a similar trend with NOX2 and ROS (Fig. 2). **B** Representative western blots and quantitative analysis of the levels of Beclin-1 and p62 and the effects of apocynin on the expression of these factors in ischemic cortical tissue at the indicated timepoints during reperfusion. **D** Quantitative analysis of Beclin-1 levels. The expression of Beclin-1 was increased 3 days poststroke but gradually decreased over time until 14 days poststroke. Apocynin downregulated Beclin-1 expression in all groups, accompanied by NOX2 and ROS decreases. **E** Quantitative analysis of levels of p62. The levels of p62 were reduced 3 days after MCAO but gradually increased with time until 14 days. Apocynin upregulated p62 expression in all groups, accompanied by NOX2 and ROS decreases. Scale bar = 50 µm. *P < 0.05 vs. the vehicle group, ^&^*P* < 0.05 vs. the sham group, ^*#*^*P* < 0.05 vs. the 3d vehicle group, ^*^*^*P* < 0.05 vs. the 7d vehicle group. All data are expressed as the mean ± SD and were analyzed by one-way ANOVA with Bonferroni’s post hoc test. There were 5 animals in each group

Autophagy, which is the intracellular mechanism by which cells digest and recycle unfolded proteins and dysfunctional organelles, is emerging as a major target of ROS and NOX enzymes [34, 35]. To explore the underlying mechanisms of the biphasic effects of ROS in the development of ischemic stroke, we focused on autophagy. The western blot results showed that the autophagy-related protein Beclin-1 was similar to NOX2 and ROS and significantly increased on Day 3 after stroke but gradually decreased between Day 3 and Day 14, and this effect was significantly inhibited by the NOX2 inhibitor apocynin (Fig. 5). Moreover, p62/SQSTM1 is an autophagosome adapter protein that is degraded by autophagy, whereas p62 accumulates when autophagy is inhibited. We also measured the expression of p62 by Western blotting, and the results showed that the levels of p62 were opposite to those of NOX2 and ROS, decreasing on Day 3 after stroke but gradually increasing between Day 3 and Day 14 (Fig. 5). We then measured the expression of another autophagy-related protein, LC3B, by immunofluorescent staining and confocal microscopy. The results suggested that LC3B was dramatically increased in the ischemic brain during the early stage of stroke but slightly increased during the late stage, which was also similar to the trend of NOX2/ROS and was inhibited by apocynin administration. These findings suggest that autophagy after stroke is likely dependent on NOX2/ROS activity.

### Apocynin exacerbates the inflammatory response by inhibiting autophagy 7 and 14 days after stroke

**Fig. 6:**
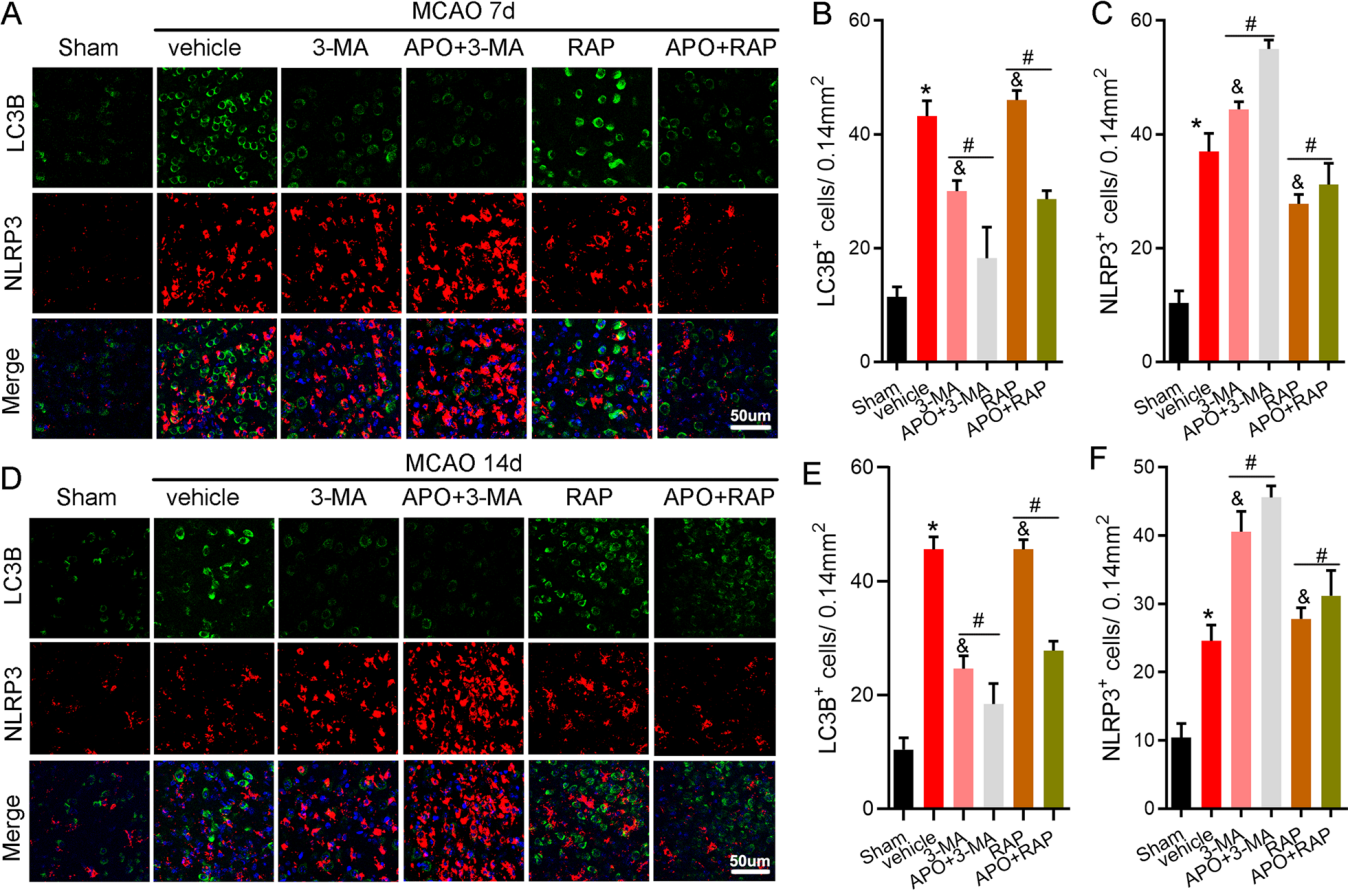
Apocynin activated the NLRP3 inflammasome via autophagy 7 and 14 days after stroke. During the delayed stage of ischemia, rapamycin induced autophagy but inactivated the NLPR3^+^ inflammasome, and 3-MA inhibited autophagy but activated the expression of NLRP3. Apocynin plus 3-MA or rapamycin inhibited autophagy but promoted NLPR3^+^ inflammasome activation. **A** Representative immunofluorescence images of colocalized NLPR3 and LC3B staining and DAPI counterstaining 7 days after stroke in the ischemic border. **B**, **C** Quantification of NLRP3- and LC3B-positive cells 7 days after stroke. **D** Representative immunofluorescence images of colocalized NLRP3 and LC3B staining and DAPI counterstaining 14 days after stroke in the ischemic border. **E**, **F** Quantification of NLRP3- and LC3B-positive cells 14 days after stroke. Scale bar = 50 μm. *P < 0.05 vs. the sham group, ^&^*P* < 0.05 vs. the vehicle group, ^#^*P* < 0.05 vs. the APO group. All data are expressed as the mean ± SD. *n* = 5/group

**Fig. 7:**
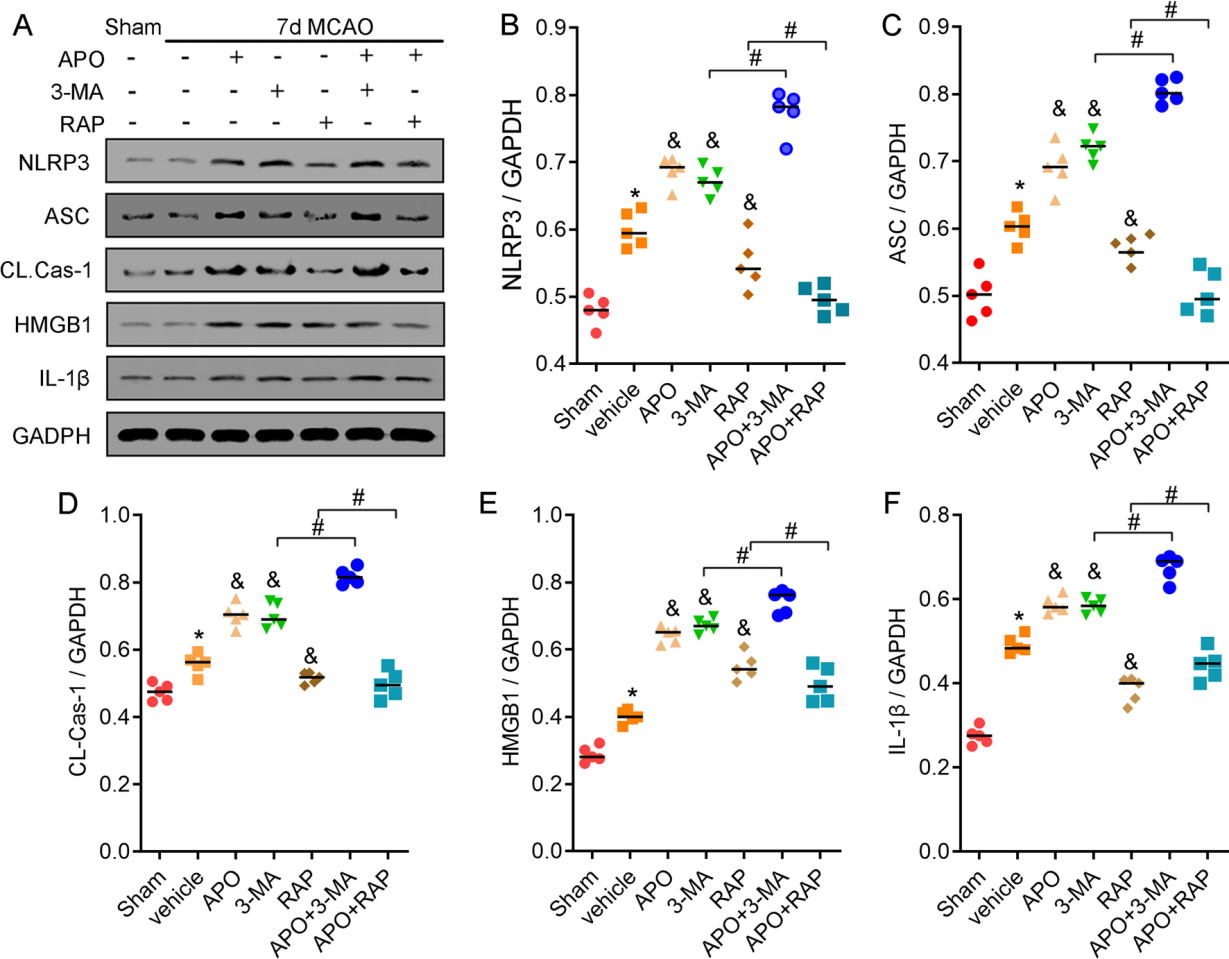
Apocynin increased the expression of NLRP3 inflammasome-associated proteins via autophagy 7 days after stroke. **A** Representative western blot showing that rapamycin inactivated, while 3-MA promoted, the expression of NLRP3-associated proteins, including NLRP3, ASC, CL-caspase-1, HMGB1 and IL-1β, in ischemic cortical tissue after 7 days of reperfusion. Apocynin plus 3-MA or rapamycin increased or maintained the expression of NLRP3 inflammasome-associated proteins. **B**–**F** Quantitative analysis of the protein levels of NLRP3, ASC, CL-caspase-1, HMGB1 and IL-1β in ischemic cortical tissue. *P < 0.05 vs. the sham group, ^&^*P* < 0.05 vs. the vehicle group, ^#^*P* < 0.05 vs. the APO group. All data are expressed as the mean ± SD. *n* = 5/group

As an intracellular bulk degradation system, autophagy contributes to pathologies associated with neuroinflammation regulation in mouse models of ischemic stroke [36, 37]. We then hypothesized that NOX2-derived ROS resolve inflammation during the delayed phase of stroke by activating autophagy. We used the autophagy inducer rapamycin and the autophagy inhibitor 3-methyladenine (3-MA) to induce and inhibit autophagy, respectively. The confocal immunostaining results showed that rapamycin induced autophagy but decreased the number of NLPR3^+^ cells; however, 3-MA inhibited autophagy activation and increased NLRP3 expression on Days 7 and 14 after MCAO (Fig. 6A). To study whether NOX2 inhibition regulates NLRP3 expression via autophagy inhibition, apocynin was injected in combination with either 3-MA or rapamycin. Confocal microscopic analysis showed that apocynin increased NLRP3^+^ cell numbers in the groups treated with apocynin plus 3-MA and rapamycin at 7 and 14 days after stroke compared with rapamycin or 3-MA alone (Fig. 6A). In addition, the western blot results indicated that apocynin promoted the expression of NLRP3 inflammasome-associated proteins, including NLRP3, ASC, CL-caspase-1 and IL-1β, in animals treated with the autophagy inhibitor 3-MA (Fig. 7). In contrast, apocynin did not significantly change the expression of NLRP3 inflammasome-associated proteins (Fig. 7) in mice treated with the autophagy agonist rapamycin. These results strongly indicate that apocynin exacerbates the inflammatory response by inhibiting autophagy.

### NOX2 inhibition by apocynin attenuates angiogenesis by inhibiting autophagy 7 and 14 days after stroke

**Fig. 8:**
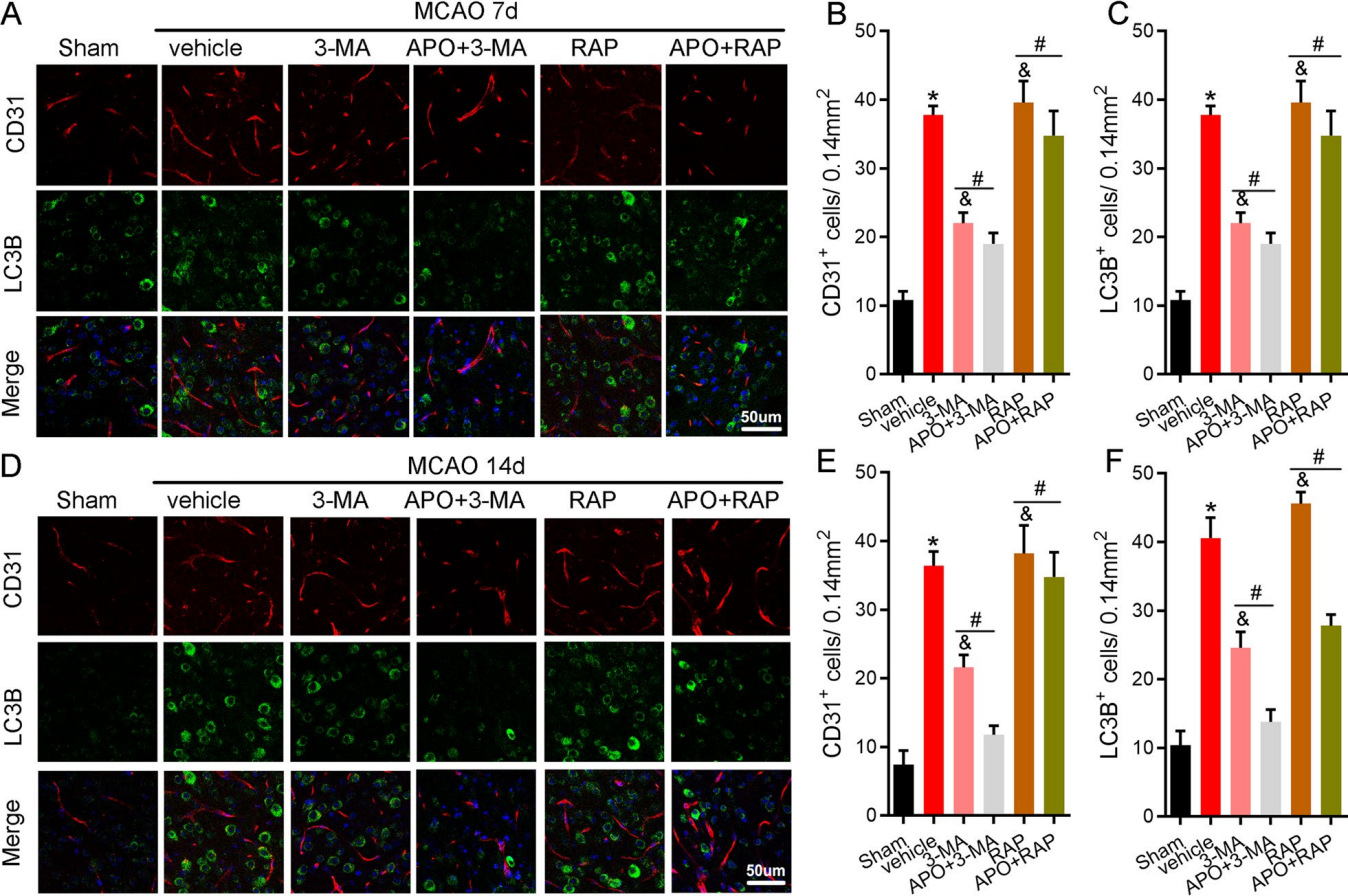
Apocynin inhibited angiogenesis via autophagy 7 and 14 days after stroke. At the delayed stage of ischemia, rapamycin induced autophagy and promoted angiogenesis, and 3-MA inhibited autophagy and reduced the number of CD31^+^ stained cells. Apocynin plus 3-MA or rapamycin resulted in autophagy inhibition and simultaneously suppressed angiogenesis compared with 3-MA or rapamycin only. **A** Representative immunofluorescence images of colocalized CD31 and LC3B staining and DAPI counterstaining 7 days after stroke in the ischemic border. **B**, **C** Quantification of CD31- and LC3B-positive cells at 7 days after stroke. **D** Representative immunofluorescence images of colocalized CD31 and LC3B staining and DAPI counterstaining 14 days after stroke in the ischemic border. **E**, **F** Quantification of CD31- and LC3B-positive cells at 14 days after stroke. Scale bar = 50 μm. *P < 0.05 vs. the sham group, ^&^*P* < 0.05 vs. the vehicle group, ^#^*P* < 0.05 vs. the APO group. All data are expressed as the mean ± SD. *n* = 5/group

**Fig. 9:**
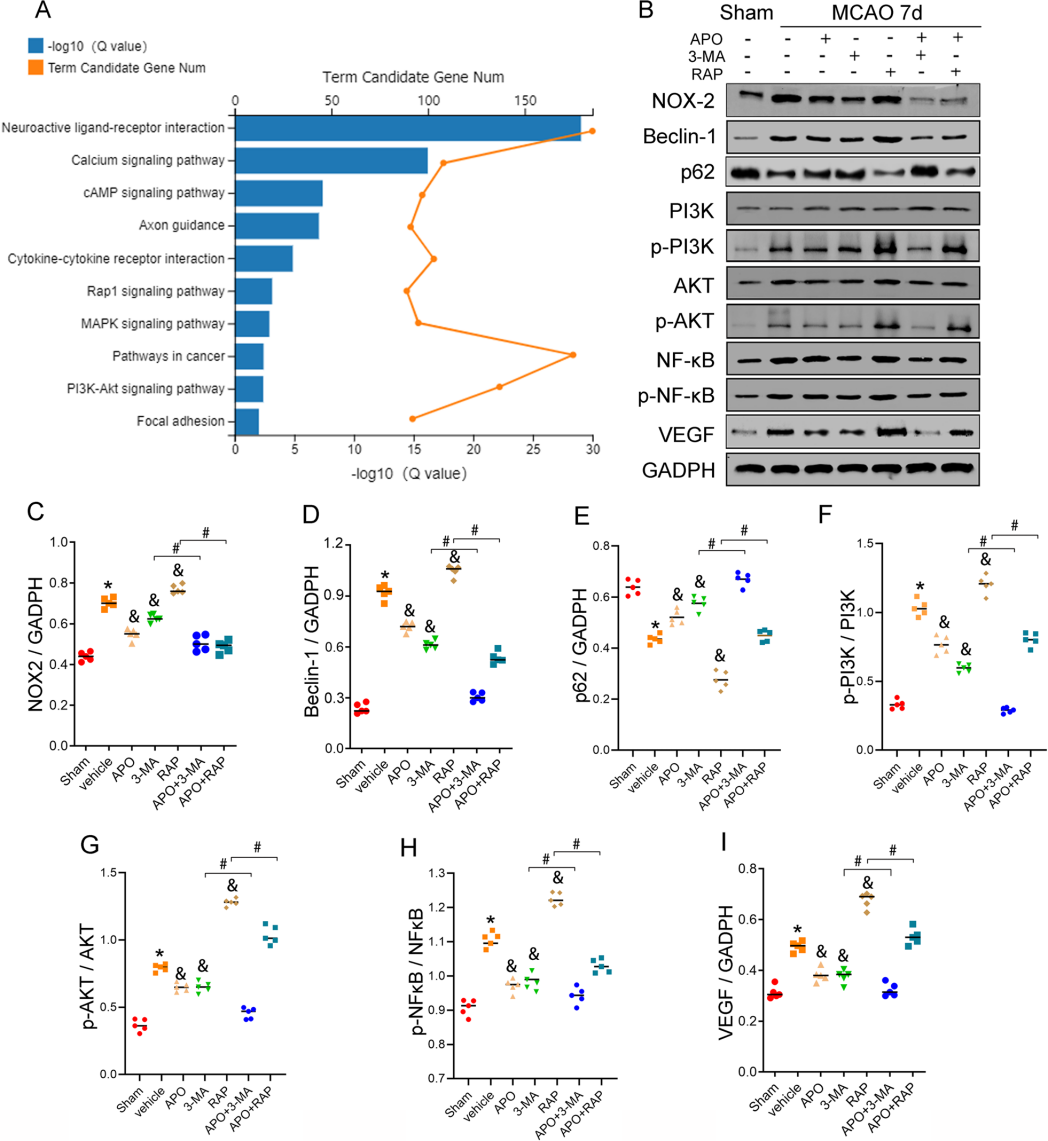
Involvement of PI3K/Akt/NF-kB signaling in autophagy-induced angiogenesis 7 days after stroke. **A** KEGG pathway analysis of the genes in the apocynin group vs. the vehicle group compared with the vehicle group vs. the sham group. The top 10 pathways are shown. **B** Western blot analysis showing that inhibition of NOX2 by apocynin decreased the expression of PI3K/Akt/NF-kB signaling pathway proteins, including PI3K, p-PI3K, Akt, p-Akt, NF-κB p65 and p-NF-κB p65, as well as VEGF, and downregulated the autophagy proteins Beclin-1 and p62 in ischemic cortical tissue after 7 days of reperfusion. **C**–**I** Quantitative analysis of the protein levels of NOX2, Beclin-1, p62, p-PI3K, p-Akt, p-NF-κB p65 and VEGF in ischemic cortical tissue. *P < 0.05 vs. the sham group, ^*&*^*P* < 0.05 vs. the vehicle group, ^*#*^*P* < 0.05 vs. the APO group. All data are expressed as the mean ± SD. *n* = 5/group

Autophagy plays an important role in angiogenesis. We thus investigated whether the NOX2/ROS pathway enhances angiogenesis by modulating autophagy during brain recovery after stroke. The confocal microscopy results suggested that autophagy inhibition by 3-MA reduced staining of the angiogenesis marker CD31. However, rapamycin treatment significantly increased CD31 staining on day 7 and 14 following stroke (Fig. 8), and this effect was reduced by apocynin. We further found that apocynin inhibited VEGF expression in animals treated with rapamycin (Fig. 9). Consistently, apocynin further inhibited the expression of CD31 and VEGF 7 and 14 days following stroke in mice that received 3-MA (Fig. 9). Taken together, these results suggest that apocynin inhibits angiogenesis by inhibiting autophagy.

### PI3K/Akt/NF-kB signaling is involved in NOX2–ROS-dependent autophagy-induced angiogenesis and anti-inflammatory effects during the long-term recovery of ischemic stroke

RNA sequencing and KEGG pathway analysis indicated that genes related to the NF-kappa B signaling pathway, NOD-like receptor signaling pathway and Toll-like receptor signaling pathway were enriched at 7 days after MCAO (Additional file 5: Fig. S5 and Additional file 6: Fig. S6). After apocynin treatment, the differentially upregulated genes were enriched in the PI3K–Akt signaling pathway, NF-kappa B signaling pathway and MAPK signaling pathway, as shown by KEGG pathway analysis. Next, the two groups of differentially expressed genes were further compared, and the results showed that apocynin treatment resulted in the upregulated genes being enriched in several key signaling pathways, including the PI3K–Akt signaling pathway, cytokine–cytokine receptor interactions and the N-Fkappa B signaling pathway (Fig. 9A). These findings indicated that the PI3K–Akt signaling pathway may participate in NOX2-mediated tissue recovery during the long-term stage of brain ischemia. The PI3K/Akt/NF-kB pathway is well known to be involved in autophagy-dependent responses to angiogenesis and inflammation inhibition. In addition, to further confirm whether NOX2 modulates these processes via the PI3K–Akt signaling pathway during the long-term recovery of ischemic stroke, we assessed the levels of pathway-related proteins by western blot analysis. We found that rapamycin promoted the protein expression of phosphorylated PI3K, Akt, NF-kB and VEGF, downregulated the autophagy-associated protein Beclin-1 and upregulated p62, while 3-MA inhibited expression of these factors (except p62) compared with the vehicle (Fig. 9B). However, apocynin reduced the levels of these protein that were increased by rapamycin and further attenuated the levels of proteins that were already reduced by 3-MA (Fig. 9). Figures 7 and 8 show that the activation of the NLRP3 inflammasome and its associated proteins, including NLRP3, ASC, CL-caspase-1, HMGB1 and IL-1β, was inhibited by apocynin treatment alone or combined with 3-MA or rapamycin. Therefore, combining Fig. 9 with Figs. 7 and 8, our results suggest that the PI3K/Akt/NF-kB signaling pathway is involved in angiogenesis and inflammation inhibition induced by NOX–ROS-dependent autophagy during the recovery stage of ischemic stroke.

## Discussion

While the role of NOX2-derived ROS in the brain remains controversial and has been suggested to be deleterious in some studies, recent evidence has emerged to support a neuroprotective role of this factor [14, 38–40]. The present study was the first to present novel results that NOX2 plays opposing roles in the acute and chronic phases of ischemic stroke by using the NOX2 inhibitor apocynin. While NOX2 inhibition attenuated acute infarction and improved neurological scores and behavioral performance on Day 3 poststroke, it blocked the functional recovery from 7 to 14 days. We found that ROS production increased after stroke in a NOX2-dependent manner, indicating that NOX2 is a major source of ROS after MCAO. Similarly, we demonstrated that the NOX2 inhibitor apocynin increased angiogenesis and reduced the NLRP3 inflammasome in the acute phase of stroke but suppressed angiogenesis and promoted NLRP3 inflammasome activation in the later stage of stroke. Then, by using a specific autophagy inhibitor and inducer, we provided evidence that autophagy, which is also a double-edged sword, is involved in NLRP3 inflammasome activation and the promotion of angiogenesis induced by NOX2–ROS during recovery. Thus, we have demonstrated that angiogenesis and anti-inflammatory effects were enhanced by NOX2/ROS-induced autophagy through phosphorylation of the PI3K/Akt/NF-kB signaling pathway. To the best of our knowledge, this is the first study to demonstrate that NOX2 has distinctive effects during different phases after ischemic stroke. These novel findings provide insights for re-evaluating the role of ROS in brain injury and functional recovery after stroke.

Our results regarding the dual roles of NOX2 in stroke are supported by previous studies. First, the contribution of NOX2/ROS to ischemic brain damage during the acute period of stroke is well known [3, 41–43]. It has been well established that the abrupt overproduction of ROS after ischemia/reperfusion exacerbates acute brain injury. NOX2 is the classic phagocytic NOX, and its primary role is the generation of free radicals, especially ROS, during ischemic stroke [22, 44]. Our results showed that the production of NOX2 and ROS were both significantly increased 3 days after stroke and gradually decreased to the levels in the sham group on Day 14. Apocynin, a NOX2 inhibitor, decreased NOX2 expression and reduced ROS production. Therefore, it is reasonable to conclude that NOX2 inhibition attenuates acute brain injury by blocking ROS production. Second, the dual effects of ROS have been observed in other models, as excessive ROS cause detrimental effects and physiological levels of ROS result in benefits for the ischemic brain [45, 46]. In addition, another study suggests that ROS exert biphasic effects on the ischemic brain, and the acute damaging effect occurs during the early phase, while the beneficial effects on neurovascular remodeling and functional restoration occur during the recovery stage [23]. In our current study, we found that ROS are NOX2-dependent, and inhibiting NOX2 with apocynin improved brain damage, reduced neurological deficiencies and increased mouse survival on Day 3 after MCAO. In addition, we found that NOX2 inhibition in the ischemic brain by apocynin resulted in increased mortality and reduced functional recovery on Days 7 and 14 after stroke. Our results suggested the dual effects of NOX2–ROS during the different stages of brain ischemia, which were not only consistent with the theory regarding the biphasic roles of ROS but also provided solid evidence for this conclusion.

Our findings demonstrated that the underlying pathological mechanism by which NOX-2 mediates brain injury and functional recovery is linked to neuroinflammation and angiogenesis. The NLRP3 inflammasome, which is the best characterized inflammasome to date, contributes significantly to brain injury and neuroinflammation after stroke [22]. The inflammasome can be activated by many factors, including environmental irritants, endogenous danger signals, pathogens, and various pathogen-associated molecular patterns (PAMPs). Substantial evidence indicates that ROS are proximal signals for NLRP3 inflammasome activation in CIRI [47]. ROS promote tissue inflammation and activate the immune response through the NLRP3 inflammasome during ischemia/reperfusion [48]. To confirm whether the ROS-induced NLRP3 inflammasome contributes to the dual functions of NOX2, we evaluated the dynamic changes in the NLRP3 inflammasome after MCAO and found that the number of NLRP3^+^ cells increased on Day 3 but gradually decreased thereafter. However, the NOX2 inhibitor apocynin reduced NLRP3^+^ cells on Day 3 but promoted inflammation during brain functional restoration, suggesting a critical role of NOX2 in the neuroinflammatory response. In contrast with neuroinflammation, angiogenesis promotes cell repair and survival after stroke. In the recovery phase of stroke, the generation of ROS-dependent growth factors promotes angiogenesis and induces the proliferation and differentiation of vascular smooth muscle cells to affect vascular remodeling [49]. In addition, NOX2 has been shown to be advantageous in vascular proliferation after hind limb ischemic injury [7, 50]. Moreover, NOX2 was expressed on new blood vessels in the rat brain 7 days after stroke [13, 51]. In the current study, we found that the expression of the blood vessel marker CD31 was reduced on Day 3 but gradually increased at 7–14 days, and apocynin enhanced CD31 levels on Day 3 but inhibited its expression during brain recovery.

We further hypothesized that autophagy is involved in the effects of NOX2 on brain injury and recovery and investigated these events. As an intracellular bulk degradation system that exists ubiquitously in eukaryotes, autophagy also plays dual roles in CIRI [15]. Previous studies have suggested that oxidative stress initiates autophagosome formation and increases autophagic flux after ischemia/reperfusion [52–54]. In turn, autophagic activity could inhibit the activation of NLRP3 inflammasomes in CIRI [55]. Autophagosomes recognize the NLPR3 inflammasome through selective autophagy receptors and remove ubiquitylated inflammasomes or eliminate activators, including defective organelles, damaged DNA and mitochondrial ROS, to negatively activate the NLPR3 inflammasome [20, 56]. In contrast, autophagy inhibition or deficiency results in the activation of the NLPR3 inflammasome [19]. In the current study, we showed that the expression of autophagy-related proteins was increased along with NOX2 and ROS, and the expression of these factors was downregulated by apocynin. Consistently, the specific autophagy inhibitor 3-MA and the autophagy inducer rapamycin blocked or activated autophagy and promoted or suppressed the NLPR3 inflammasome, respectively. In addition, apocynin blocked autophagy while enhancing NLPR3 inflammasome expression compared to 3-MA or rapamycin 7 and 14 days poststroke. These results strongly indicate that NOX2/ROS inhibit inflammation through autophagy activation, playing a neuroprotective role during the delayed stage of stroke.

Autophagy is also coupled with angiogenesis [57], and it is involved in the development and fundamental roles of vascular endothelial cells [58]. This autophagy-initiated angiogenesis is mediated by ROS [21] and is a key repair mechanism for brain recovery after stroke. Insufficient autophagy is a detrimental factor that induces EC phenotypic senescence and dysfunction [59]. Thus, manipulating autophagy by genetic methods or stimulators/inhibitors can modulate angiogenesis. Consistent with these previous studies, our current study confirmed that NOX2-derived ROS-dependent autophagy was involved in regulating angiogenesis and preventing brain injury, thus accelerating brain recovery after stroke.

The molecular mechanisms by which NOX2–ROS-autophagy influences vascular proliferation and NLRP3 inflammasome inhibition are complex and poorly understood. To further explore the molecular mechanisms, we used high-throughput RNA sequencing to analyze the effect of NOX2–ROS on the ischemic brain during the recovery stage. The results indicated that the PI3K–Akt signaling pathway was enriched in the apocynin treatment group 7 days after MCAO, which indicated that this pathway might be involved in NOX2–ROS-mediated long-term recovery after ischemic insult. Moreover, the regulation of angiogenesis and the NLRP3 inflammasome is well known to be linked with the PI3K/Akt pathway. PI3K/Akt activation upregulates VEGF expression and accelerates blood vessel regeneration, while pharmacological inhibition of the PI3K/Akt signaling pathway decreases VEGF secretion and inhibits angiogenesis [60]. However, whether angiogenesis and inhibition of the NLRP3 inflammasome induced by NOX2/ROS/autophagy is mediated by the PI3K/Akt pathway remains unknown. In the current study, we confirmed the RNA sequencing results and demonstrated that the NOX2 inhibitor apocynin and the autophagy inhibitor 3-MA inhibited the protein expression of VEGF, phosphorylated PI3K and Akt, and NF-kB while promoting the expression of NLRP3 inflammasome-related proteins. In addition, VEGF and phosphorylated PI3K/Akt/NF-kB were synchronously upregulated, but NLRP3 inflammasome-related proteins were downregulated with increasing NOX2 and autophagy. Therefore, our results suggest that the PI3K/Akt/NF-kB signaling pathway is involved in angiogenesis and inhibiting inflammation induced by NOX–ROS-dependent autophagy after stroke. Our finding is consistent with the study by Du showing that autophagy triggered angiogenesis through VEGF and Akt [61]. We are aware of other conflicting findings that autophagy blocks PI3K/Akt activation and exerts antiangiogenic effects on neuroblastoma cells [62]. Although these discrepancies may be dependent on different cell types and cellular demands [58], further research is needed to clarify autophagy-induced angiogenesis and the underlying mechanisms related to the PI3K/Akt/NF-kB signaling pathway after ischemic stroke.

However, there are a number of limitations in our current study, which need to be addressed by further comprehensive investigations. First, the standard between high and low ROS levels was missing, and we only measured different levels and analyzed the statistical significance of ROS in the current study. Second, the molecular mechanism by which NOX/ROS induce autophagy after stroke is not yet known and needs to be further screened by new technologies, such as transcriptome or proteome analysis, verified and clarified.

## Conclusion

**Fig. 10:**
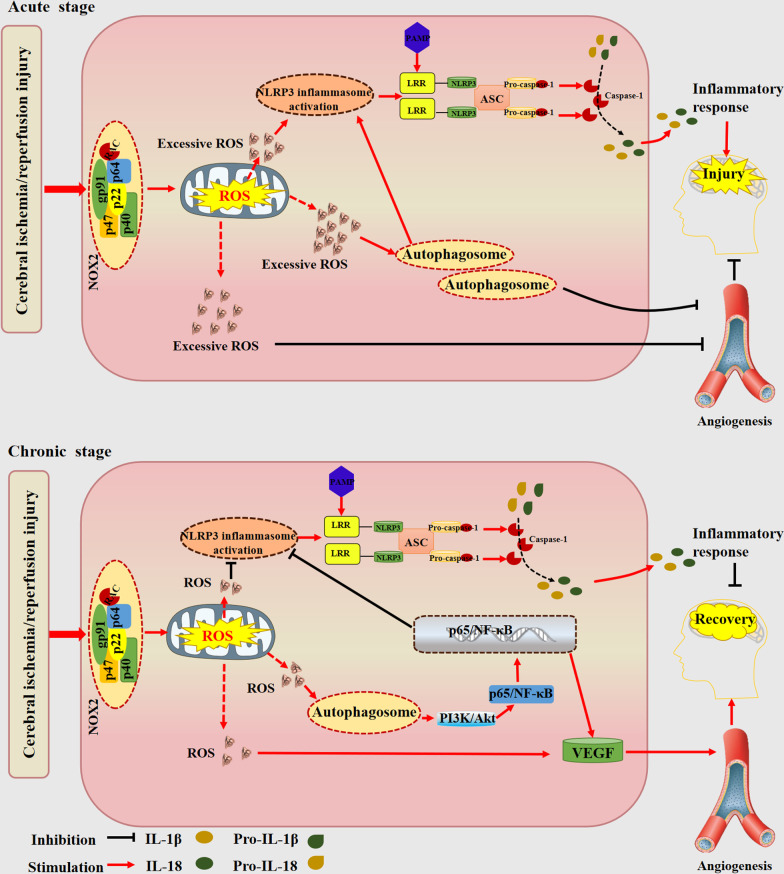
NOX2–ROS is a double-edged sword in focal cerebral ischemic stroke. During the early stage of reperfusion, NADPH oxidase 2 is extensively activated, resulting in excessive ROS production. Excessive ROS cause overgeneration of autophagosomes, which on the one hand induces activation of the NLRP3 inflammasome and mediates the inflammatory response. On the other hand, excessive ROS-dependent autophagy inhibits ischemic stroke-induced vascular growth. These processes aggravate stroke-induced brain damage. However, during the delayed stage of ischemic stroke, low levels of NOX2-induced ROS function as signaling molecules, and they can stimulate low levels of autophagosomes that inhibit NLRP3 inflammasome activation and promote vessel formation, which may be achieved through the activation of the PI3K/Akt/NF-kB signaling pathway, thus alleviating the inflammatory response and facilitating angiogenesis to exert protective effects on the ischemic brain

NOX2-derived ROS are a double-edged sword that have distinctive effects during the acute and delayed phases after stroke. NOX2 exacerbates acute brain injury after stroke by activating the NLRP3 inflammasome and blocking angiogenesis through the excessive production of ROS followed by excessive autophagy activation, but it repairs the brain during the delayed stage by generating a small quantity of ROS, inhibiting the NLRP3 inflammasome and triggering angiogenesis via a low level of autophagy activation, which is associated with the PI3K/Akt/NF-kB signaling pathway (Fig. 10).

## Supplementary Information

**Additional file 1: **Figure S1. Identification of ischemic penumbra. (A) Schematic cuts of whole brain. (B) Separation of penumbra and core on representative cresyl violet staining section.

**Additional file 2: **Figure S2. Apocynin reduces infarct size and ischemic stroke injury. (A) Infarct volume of the ipsilateral hemisphere was measured 3 days poststroke by using TTC staining. (B) Quantification of infarct volume after Apocynin treatment 3 days poststroke. Mean ± SD. *n* = 5/group. ^*^P < 0.05 vs. 0 mg/kg APO; ^*#*^* P* < 0.05 vs. 1.5 mg/kg APO.

**Additional file 3: **Figure S3. Functional classification and Gene Ontology analysis of ischemic stroke. (A) GO enrichment analysis of the differentially expressed genes that were regulated in the sham vs. vehicle groups. (B) The top 20 enriched biological processes, (C) cellular components, and (D) molecular functions are shown.

**Additional file 4: **Figure S4. Functional classification and Gene Ontology analysis of the effects of Apocynin administration on ischemic stroke. (A) GO enrichment analysis of the differentially expressed genes that were regulated in the vehicle vs. APO groups. The top 20 enriched biological processes (B), cellular components (C), and molecular functions (D) are shown.

**Additional file 5: **Figure S5. Apocynin administration alters the gene expression profile in ischemic stroke. (A) Circle plot showing that the upregulated genes were closely related to several biological processes in the sham vs. vehicle groups (B) and the vehicle vs. APO groups.

**Additional file 6: **Figure S6. KEGG pathway analysis of NOX2-downregulated genes 7 days after MCAO. (A) The top 20 pathways are shown in the sham group compared to the vehicle group and (B) the vehicle group compared to the APO group.

## Data Availability

The datasets during and/or analyzed during the current study are available from the corresponding author on reasonable request.

## Abbreviations

ROS: Reactive oxygen species
NADPH oxidase: Nicotinamide adenine dinucleotide phosphate oxidase
NOX2: NADPH oxidase 2
MCAO: Middle cerebral artery occlusion
DHE: Dihydroethidium
DEGs: Differentially expressed genes
WT: Wild-type
KEGG: Kyoto Encyclopedia of Genes and Genomes
NLRP3: NOD-like receptor protein 3
CNS: Central nervous system
CIRI: Cerebral ischemia/reperfusion injury
3-MA: 3-Methyladenine
GO analysis: Gene Ontology analysis
PI3K: Phosphatidylinositol 3 kinase
NF-kB: Nuclear factor-k-gene binding
VEGF: Vascular endothelial growth factor
PAMPs: Pathogen-associated molecular patterns

## References

[1] Radermacher KA, Wingler K, Langhauser F, Altenhofer S, Kleikers P, Hermans JJ (2013). Neuroprotection after stroke by targeting nox4 as a source of oxidative stress. Antioxid Redox Signal.

[2] Yoshioka H, Niizuma K, Katsu M, Okami N, Sakata H, Kim GS (2011). Nadph oxidase mediates striatal neuronal injury after transient global cerebral ischemia. J Cereb Blood Flow Metab.

[3] Ma MW, Wang J, Zhang Q, Wang R, Dhandapani KM, Vadlamudi RK (2017). Nadph oxidase in brain injury and neurodegenerative disorders. Mol Neurodegener.

[4] Zhang HF, Li TB, Liu B, Lou Z, Zhang JJ, Peng JJ (2015). Inhibition of myosin light chain kinase reduces nadph oxidase-mediated oxidative injury in rat brain following cerebral ischemia/reperfusion. Naunyn Schmiedebergs Arch Pharmacol.

[5] Walder CE, Green SP, Darbonne WC, Mathias J, Rae J, Dinauer MC (1997). Ischemic stroke injury is reduced in mice lacking a functional nadph oxidase. Stroke.

[6] Doverhag C, Keller M, Karlsson A, Hedtjarn M, Nilsson U, Kapeller E (2008). Pharmacological and genetic inhibition of nadph oxidase does not reduce brain damage in different models of perinatal brain injury in newborn mice. Neurobiol Dis.

[7] Urao N, Inomata H, Razvi M, Kim HW, Wary K, McKinney R (2008). Role of nox2-based nadph oxidase in bone marrow and progenitor cell function involved in neovascularization induced by hindlimb ischemia. Circ Res.

[8] Stancu IC, Cremers N, Vanrusselt H, Couturier J, Vanoosthuyse A, Kessels S (2019). Aggregated tau activates nlrp3-asc inflammasome exacerbating exogenously seeded and non-exogenously seeded tau pathology in vivo. Acta Neuropathol.

[9] He XF, Zeng YX, Li G, Feng YK, Wu C, Liang FY (2019). Extracellular asc exacerbated the recurrent ischemic stroke in an nlrp3-dependent manner. J Cereb Blood Flow Metab.

[10] Fann DY, Lee SY, Manzanero S, Tang SC, Gelderblom M, Chunduri P (2013). Intravenous immunoglobulin suppresses nlrp1 and nlrp3 inflammasome-mediated neuronal death in ischemic stroke. Cell Death Dis.

[11] Yang F, Wang Z, Wei X, Han H, Meng X, Zhang Y (2014). Nlrp3 deficiency ameliorates neurovascular damage in experimental ischemic stroke. J Cereb Blood Flow Metab.

[12] Xiong XX, Gu LJ, Shen J, Kang XH, Zheng YY, Yue SB (2014). Probenecid protects against transient focal cerebral ischemic injury by inhibiting hmgb1 release and attenuating aqp4 expression in mice. Neurochem Res.

[13] Taylor CJ, Weston RM, Dusting GJ, Roulston CL (2013). Nadph oxidase and angiogenesis following endothelin-1 induced stroke in rats: role for nox2 in brain repair. Brain Sci.

[14] Jiang F, Zhang Y, Dusting GJ (2011). Nadph oxidase-mediated redox signaling: roles in cellular stress response, stress tolerance, and tissue repair. Pharmacol Rev.

[15] Wei K, Wang P, Miao CY (2012). A double-edged sword with therapeutic potential: an updated role of autophagy in ischemic cerebral injury. CNS Neurosci Ther.

[16] Forte M, Palmerio S, Yee D, Frati G, Sciarretta S (2017). Functional role of nox4 in autophagy. Adv Exp Med Biol.

[17] Huang J, Canadien V, Lam GY, Steinberg BE, Dinauer MC, Magalhaes MA (2009). Activation of antibacterial autophagy by nadph oxidases. Proc Natl Acad Sci USA.

[18] Sciarretta S, Yee D, Ammann P, Nagarajan N, Volpe M, Frati G (2015). Role of nadph oxidase in the regulation of autophagy in cardiomyocytes. Clin Sci.

[19] Harris J, Lang T, Thomas JPW, Sukkar MB, Nabar NR, Kehrl JH (2017). Autophagy and inflammasomes. Mol Immunol.

[20] Li R, Ji Z, Qin H, Kang X, Sun B, Wang M (2014). Interference in autophagosome fusion by rare earth nanoparticles disrupts autophagic flux and regulation of an interleukin-1beta producing inflammasome. ACS Nano.

[21] Du J, Teng RJ, Guan T, Eis A, Kaul S, Konduri GG (2012). Role of autophagy in angiogenesis in aortic endothelial cells. Am J Physiol Cell Physiol.

[22] Qin YY, Li M, Feng X, Wang J, Cao L, Shen XK (2017). Combined nadph and the nox inhibitor apocynin provides greater anti-inflammatory and neuroprotective effects in a mouse model of stroke. Free Radic Biol Med.

[23] Yang J, Qi J, Xiu B, Yang B, Niu C, Yang H (2019). Reactive oxygen species play a biphasic role in brain ischemia. J Invest Surg.

[24] Wang J, Lin X, Mu Z, Shen F, Zhang L, Xie Q (2019). Rapamycin increases collateral circulation in rodent brain after focal ischemia as detected by multiple modality dynamic imaging. Theranostics.

[25] Xiong X, Xu L, Wei L, White RE, Ouyang YB, Giffard RG (2015). Il-4 is required for sex differences in vulnerability to focal ischemia in mice. Stroke.

[26] Xiong X, Gu L, Wang Y, Luo Y, Zhang H, Lee J (2016). Glycyrrhizin protects against focal cerebral ischemia via inhibition of t cell activity and hmgb1-mediated mechanisms. J Neuroinflamm.

[27] Teng F, Zhu L, Su J, Zhang X, Li N, Nie Z (2016). Neuroprotective effects of poly(adp-ribose)polymerase inhibitor olaparib in transient cerebral ischemia. Neurochem Res.

[28] Gu L, Xiong X, Zhang H, Xu B, Steinberg GK, Zhao H (2012). Distinctive effects of t cell subsets in neuronal injury induced by cocultured splenocytes in vitro and by in vivo stroke in mice. Stroke.

[29] Ashwal S, Tone B, Tian HR, Cole DJ, Pearce WJ (1998). Core and penumbral nitric oxide synthase activity during cerebral ischemia and reperfusion. Stroke.

[30] Shi M, Cao L, Cao X, Zhu M, Zhang X, Wu Z (2019). Dr-region of na(+)/k(+) atpase is a target to treat excitotoxicity and stroke. Cell Death Dis.

[31] Kang Y, Wu Z, Cai D, Lu B (2018). Evaluation of reference genes for gene expression studies in mouse and n2a cell ischemic stroke models using quantitative real-time pcr. BMC Neurosci.

[32] Symon L (1980). The relationship between cbf, evoked potentials and the clinical features in cerebral ischaemia. Acta Neurol Scand Suppl.

[33] del Zoppo GJ, Sharp FR, Heiss WD, Albers GW (2011). Heterogeneity in the penumbra. J Cereb Blood Flow Metab.

[34] Fu D, Yu JY, Yang S, Wu M, Hammad SM, Connell AR (2016). Survival or death: a dual role for autophagy in stress-induced pericyte loss in diabetic retinopathy. Diabetologia.

[35] Martinez J, Malireddi RK, Lu Q, Cunha LD, Pelletier S, Gingras S (2015). Molecular characterization of lc3-associated phagocytosis reveals distinct roles for rubicon, nox2 and autophagy proteins. Nat Cell Biol.

[36] Zheng Y, Wu Z, Yi F, Orange M, Yao M, Yang B (2018). By activating akt/enos bilobalide b inhibits autophagy and promotes angiogenesis following focal cerebral ischemia reperfusion. Cell Physiol Biochem.

[37] Zhang T, Wang H, Li Q, Huang J, Sun X (2014). Modulating autophagy affects neuroamyloidogenesis in an in vitro ischemic stroke model. Neuroscience.

[38] Bedard K, Krause KH (2007). The nox family of ros-generating nadph oxidases: physiology and pathophysiology. Physiol Rev.

[39] McCann SK, Dusting GJ, Roulston CL (2014). Nox2 knockout delays infarct progression and increases vascular recovery through angiogenesis in mice following ischaemic stroke with reperfusion. PLoS ONE.

[40] Kalogeris T, Bao Y, Korthuis RJ (2014). Mitochondrial reactive oxygen species: a double edged sword in ischemia/reperfusion vs preconditioning. Redox Biol.

[41] Chen H, Song YS, Chan PH (2009). Inhibition of nadph oxidase is neuroprotective after ischemia-reperfusion. J Cereb Blood Flow Metab.

[42] Barua S, Kim JY, Yenari MA, Lee JE (2019). The role of nox inhibitors in neurodegenerative diseases. IBRO Rep.

[43] Lou Z, Wang AP, Duan XM, Hu GH, Song GL, Zuo ML (2018). Upregulation of nox2 and nox4 mediated by tgf-beta signaling pathway exacerbates cerebral ischemia/reperfusion oxidative stress injury. Cell Physiol Biochem.

[44] Kahles T, Brandes RP (2013). Which nadph oxidase isoform is relevant for ischemic stroke? The case for nox 2. Antioxid Redox Signal.

[45] Tang X, Zhong W, Tu Q, Ding B (2014). Nadph oxidase mediates the expression of mmp-9 in cerebral tissue after ischemia-reperfusion damage. Neurol Res.

[46] Emanuele S, D'Anneo A, Calvaruso G, Cernigliaro C, Giuliano M, Lauricella M (2018). The double-edged sword profile of redox signaling: oxidative events as molecular switches in the balance between cell physiology and cancer. Chem Res Toxicol.

[47] Abderrazak A, Syrovets T, Couchie D, El Hadri K, Friguet B, Simmet T (2015). Nlrp3 inflammasome: from a danger signal sensor to a regulatory node of oxidative stress and inflammatory diseases. Redox Biol.

[48] Rubartelli A (2012). Redox control of nlrp3 inflammasome activation in health and disease. J Leukoc Biol.

[49] Abid MR, Spokes KC, Shih SC, Aird WC (2007). Nadph oxidase activity selectively modulates vascular endothelial growth factor signaling pathways. J Biol Chem.

[50] Tojo T, Ushio-Fukai M, Yamaoka-Tojo M, Ikeda S, Patrushev N, Alexander RW (2005). Role of gp91phox (nox2)-containing nad(p)h oxidase in angiogenesis in response to hindlimb ischemia. Circulation.

[51] Weston RM, Lin B, Dusting GJ, Roulston CL (2013). Targeting oxidative stress injury after ischemic stroke in conscious rats: limited benefits with apocynin highlight the need to incorporate long term recovery. Stroke Res Treat.

[52] Li L, Tan J, Miao Y, Lei P, Zhang Q (2015). Ros and autophagy: interactions and molecular regulatory mechanisms. Cell Mol Neurobiol.

[53] Chen Y, Azad MB, Gibson SB (2009). Superoxide is the major reactive oxygen species regulating autophagy. Cell Death Differ.

[54] Kaushal GP, Chandrashekar K, Juncos LA (2019). Molecular interactions between reactive oxygen species and autophagy in kidney disease. Int J Mol Sci.

[55] Wang Y, Meng C, Zhang J, Wu J, Zhao J (2019). Inhibition of gsk-3beta alleviates cerebral ischemia/reperfusion injury in rats by suppressing nlrp3 inflammasome activation through autophagy. Int Immunopharmacol.

[56] Nakahira K, Haspel JA, Rathinam VA, Lee SJ, Dolinay T, Lam HC (2011). Autophagy proteins regulate innate immune responses by inhibiting the release of mitochondrial DNA mediated by the nalp3 inflammasome. Nat Immunol.

[57] He Q, Liu Q, Chen Y, Meng J, Zou L (2018). Long-zhi decoction medicated serum promotes angiogenesis in human umbilical vein endothelial cells based on autophagy. Evid Based Complement Alternat Med.

[58] Hassanpour M, Rezabakhsh A, Pezeshkian M, Rahbarghazi R, Nouri M (2018). Distinct role of autophagy on angiogenesis: Highlights on the effect of autophagy in endothelial lineage and progenitor cells. Stem Cell Res Ther.

[59] Vion AC, Kheloufi M, Hammoutene A, Poisson J, Lasselin J, Devue C (2017). Autophagy is required for endothelial cell alignment and atheroprotection under physiological blood flow. Proc Natl Acad Sci USA.

[60] Karar J, Maity A (2011). Pi3k/akt/mtor pathway in angiogenesis. Front Mol Neurosci.

[61] Du JH, Li X, Li R, Cheng BX, Kuerbanjiang M, Ma L (2017). Role of autophagy in angiogenesis induced by a high-glucose condition in rf/6a cells. Ophthalmologica.

[62] Kim KW, Paul P, Qiao J, Lee S, Chung DH (2013). Enhanced autophagy blocks angiogenesis via degradation of gastrin-releasing peptide in neuroblastoma cells. Autophagy.

