# Supplementary figures and images for: NOX2-mediated reactive oxygen species are double-edged swords in focal cerebral ischemia in mice
Source: J Neuroinflammation. 2022 Jul 14;19:184. doi: 10.1186/s12974-022-02551-6 (PMC9281066; doi:10.1186/s12974-022-02551-6)

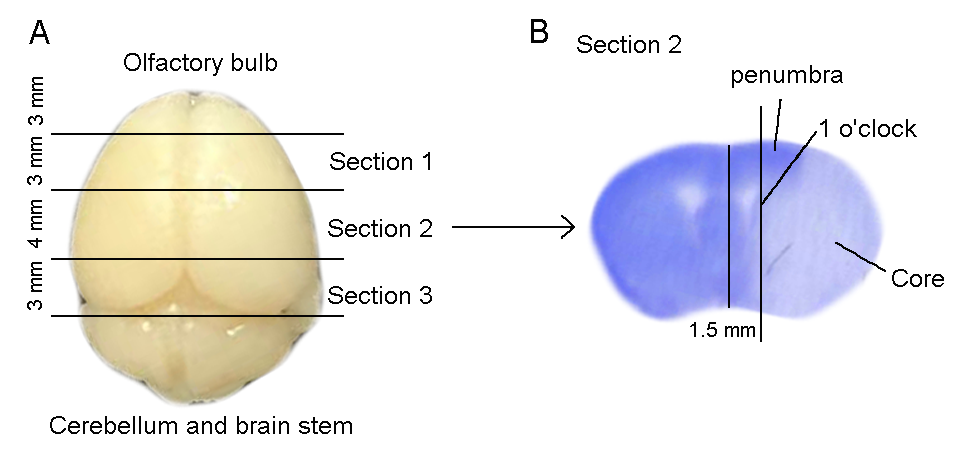

Supplement: Supplementary file 1 — Additional file 1: Figure S1. Identification of ischemic penumbra. (A) Schematic cuts of whole brain. (B) Separation of penumbra and core on representative cresyl violet staining section. [file 12974_2022_2551_MOESM1_ESM.tif]

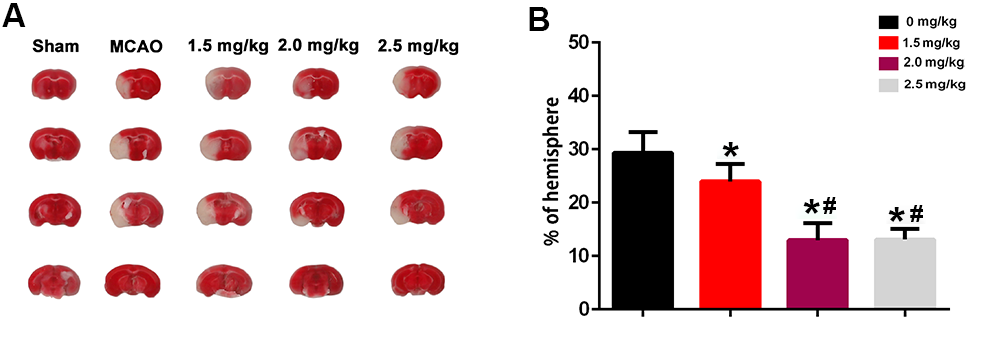

Supplement: Supplementary file 2 — Additional file 2: Figure S2. Apocynin reduces infarct size and ischemic stroke injury. (A) Infarct volume of the ipsilateral hemisphere was measured 3 days poststroke by using TTC staining. (B) Quantification of infarct volume after Apocynin treatment 3 days poststroke. Mean ± SD. n = 5/group. *P < 0.05 vs. 0 mg/kg APO; # P < 0.05 vs. 1.5 mg/kg APO. [file 12974_2022_2551_MOESM2_ESM.tif]

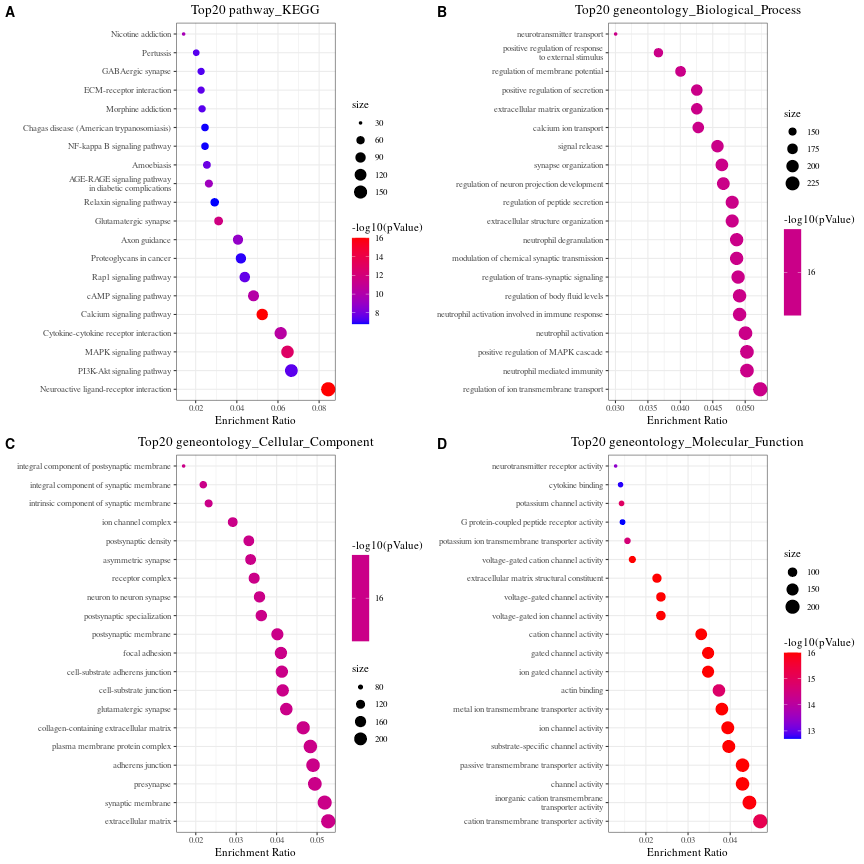

Supplement: Supplementary file 3 — Additional file 3: Figure S3. Functional classification and Gene Ontology analysis of ischemic stroke. (A) GO enrichment analysis of the differentially expressed genes that were regulated in the sham vs. vehicle groups. (B) The top 20 enriched biological processes, (C) cellular components, and (D) molecular functions are shown. [file 12974_2022_2551_MOESM3_ESM.png]

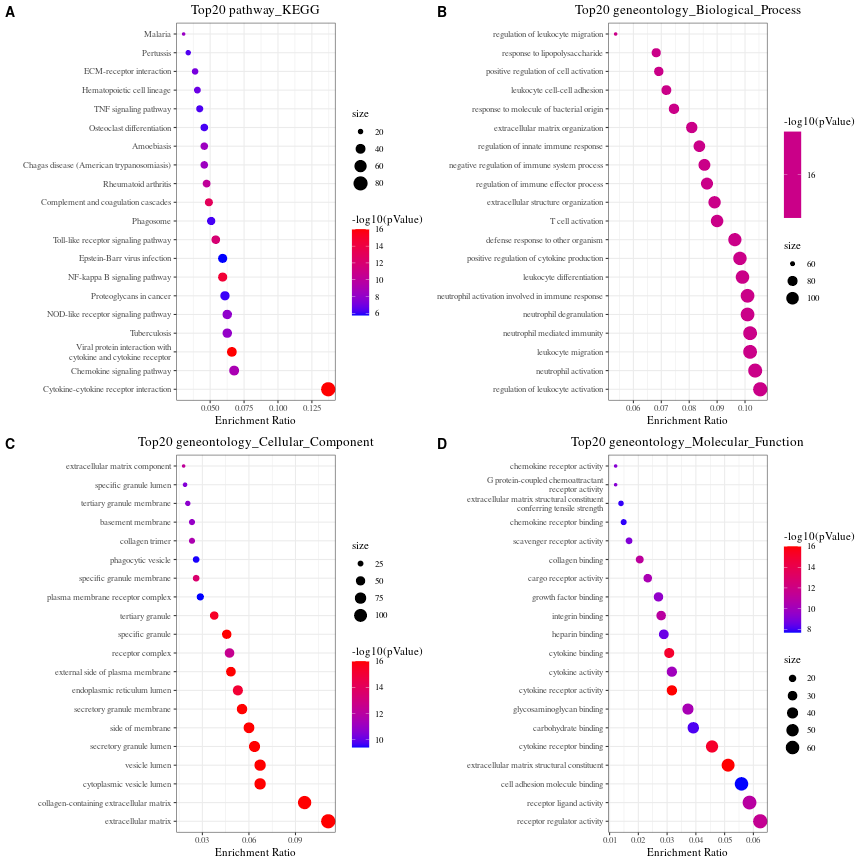

Supplement: Supplementary file 4 — Additional file 4: Figure S4. Functional classification and Gene Ontology analysis of the effects of Apocynin administration on ischemic stroke. (A) GO enrichment analysis of the differentially expressed genes that were regulated in the vehicle vs. APO groups. The top 20 enriched biological processes (B), cellular components (C), and molecular functions (D) are shown. [file 12974_2022_2551_MOESM4_ESM.png]

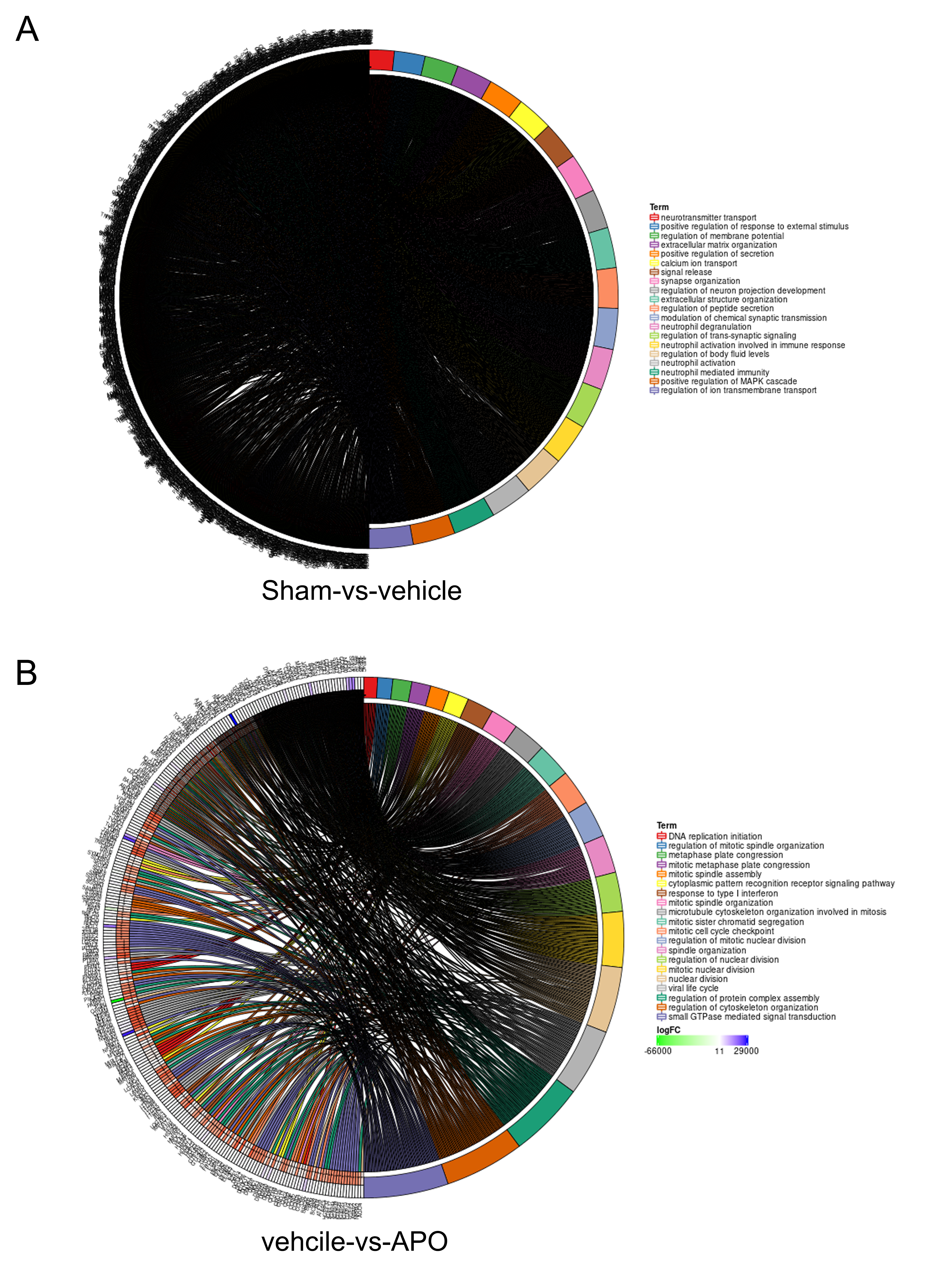

Supplement: Supplementary file 5 — Additional file 5: Figure S5. Apocynin administration alters the gene expression profile in ischemic stroke. (A) Circle plot showing that the upregulated genes were closely related to several biological processes in the sham vs. vehicle groups (B) and the vehicle vs. APO groups. [file 12974_2022_2551_MOESM5_ESM.tif]

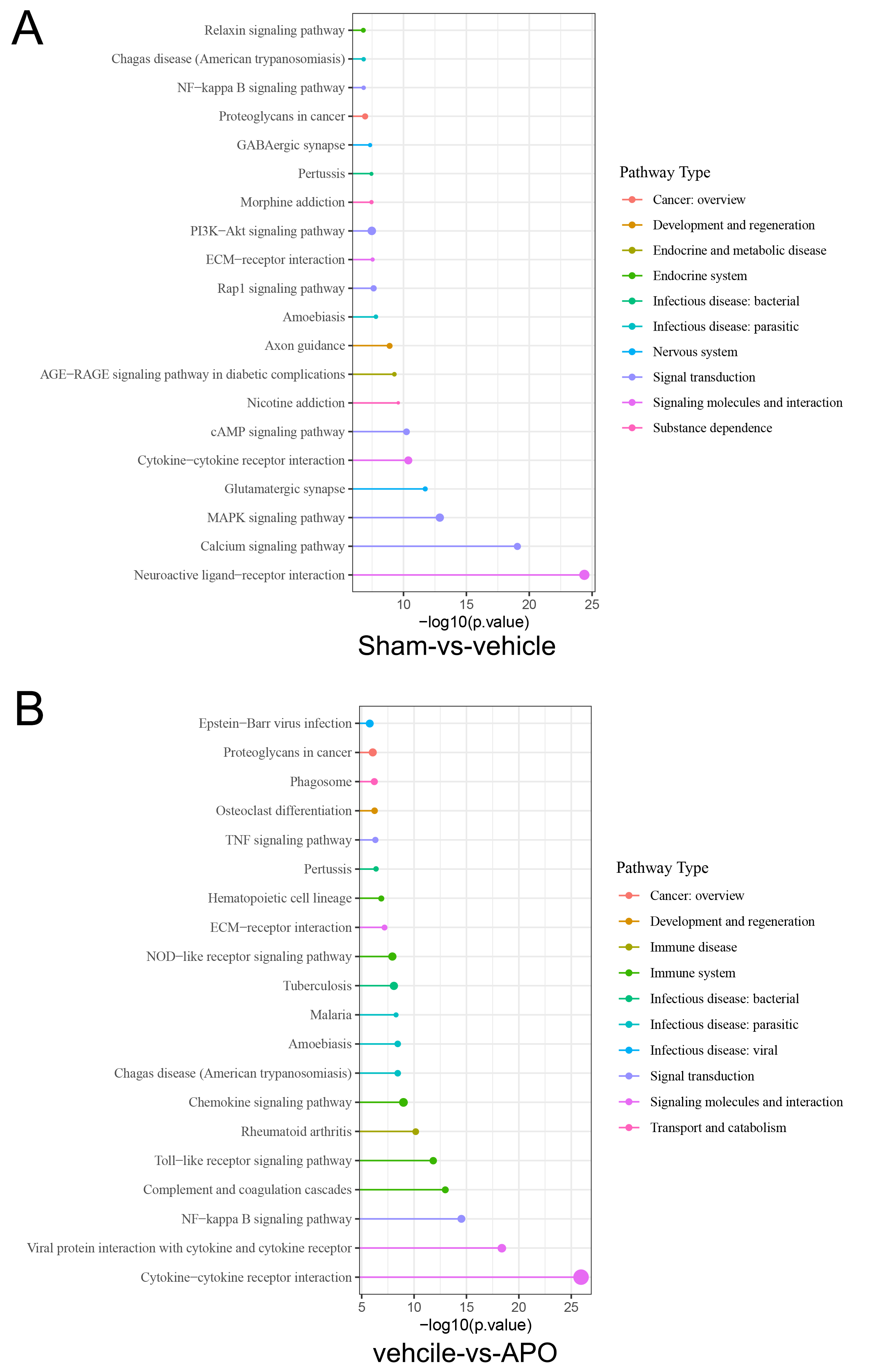

Supplement: Supplementary file 6 — Additional file 6: Figure S6. KEGG pathway analysis of NOX2-downregulated genes 7 days after MCAO. (A) The top 20 pathways are shown in the sham group compared to the vehicle group and (B) the vehicle group compared to the APO group. [file 12974_2022_2551_MOESM6_ESM.tif]
